# SHARK enables sensitive detection of evolutionary homologs and functional analogs in unalignable and disordered sequences

**DOI:** 10.1073/pnas.2401622121

**Published:** 2024-10-09

**Authors:** Chi Fung Willis Chow, Soumyadeep Ghosh, Anna Hadarovich, Agnes Toth-Petroczy

**Affiliations:** ^a^Max Planck Institute of Molecular Cell Biology and Genetics, Dresden 01307, Germany; ^b^Center for Systems Biology Dresden, Dresden 01307, Germany; ^c^Cluster of Excellence Physics of Life, Technische Universität Dresden, Dresden 01062, Germany

**Keywords:** intrinsically disordered protein regions, machine learning, homology detection, sequence to function paradigm

## Abstract

Intrinsically disordered regions (IDRs) are structurally flexible protein segments with regulatory functions in multiple contexts, such as in the assembly of biomolecular condensates. Since IDRs undergo more rapid evolution than ordered regions, identifying homology of such poorly conserved regions remains challenging for state-of-the-art alignment-based methods that rely on position-specific conservation of residues. Thus, systematic functional annotation and evolutionary analysis of IDRs have been limited, despite them comprising ~21% of proteins. To accurately assess homology between unalignable sequences, we developed an alignment-free sequence comparison algorithm, SHARK (Similarity/Homology Assessment by Relating K-mers). We trained SHARK-dive, a machine learning homology classifier, which achieved superior performance to standard alignment-based approaches in assessing evolutionary homology in unalignable sequences. Furthermore, it correctly identified dissimilar but functionally analogous IDRs in IDR-replacement experiments reported in the literature, whereas alignment-based tools were incapable of detecting such functional relationships. SHARK-dive not only predicts functionally similar IDRs at a proteome-wide scale but also identifies cryptic sequence properties and motifs that drive remote homology and analogy, thereby providing interpretable and experimentally verifiable hypotheses of the sequence determinants that underlie such relationships. SHARK-dive acts as an alternative to alignment to facilitate systematic analysis and functional annotation of the unalignable protein universe.

## Significance

Current methods of assessing the similarity between proteins are based on protein sequence alignment. However, alignment is ineffective for intrinsically disordered regions (IDRs) given their rapid evolution. Nonetheless, studies have shown that IDRs are functional and common across proteomes. They therefore represent a significant part of the protein universe which is difficult to analyze with current tools. SHARK is an alignment-free sequence comparison algorithm developed specifically for unalignable IDRs. It is further incorporated into SHARK-dive, a classifier which can outperform alignment-based methods in assessing evolutionary homology for a set of unalignable sequences, identify functional IDR analogs reported in the literature, and offer testable hypotheses of sequence regions that drive homology/analogy to aid future studies of IDRs and other unalignable regions.

A key goal in biology is to decipher the function of all proteins. Understanding of sequence–function relationships by identifying sequences/characteristics conferring a particular function would ultimately facilitate rational protein design (1). While it is impossible to experimentally annotate the function of every sequence due to the size of the protein universe (the 2022_03 release of UniProt has >227 million proteins (2)), homology-based protein function inference has aided in this effort (3–5). Specifically, sequence alignment algorithms such as Smith–Waterman local alignment (SW) (6), from which BLAST (7, 8) was developed as a faster heuristic, and hidden Markov model algorithms such as HMMer (9) achieved great success in identifying homologous sequences. To date, most conserved regions have been grouped into domain families and cataloged in databases such as Pfam, where the function of the member sequences of a family is predicted to be similar (10, 11). Nevertheless, a significant fraction of residues cannot be easily aligned to domain families (henceforth referred to as unalignable sequences). Recent Pfam releases have indicated a plateau in the percentage of UniProt sequences (~73%) and residues (~53%) mapped to domains (10), suggesting that a large proportion of sequences and residues is and will remain challenging for alignment-based analyses.

**Fig. 1.**
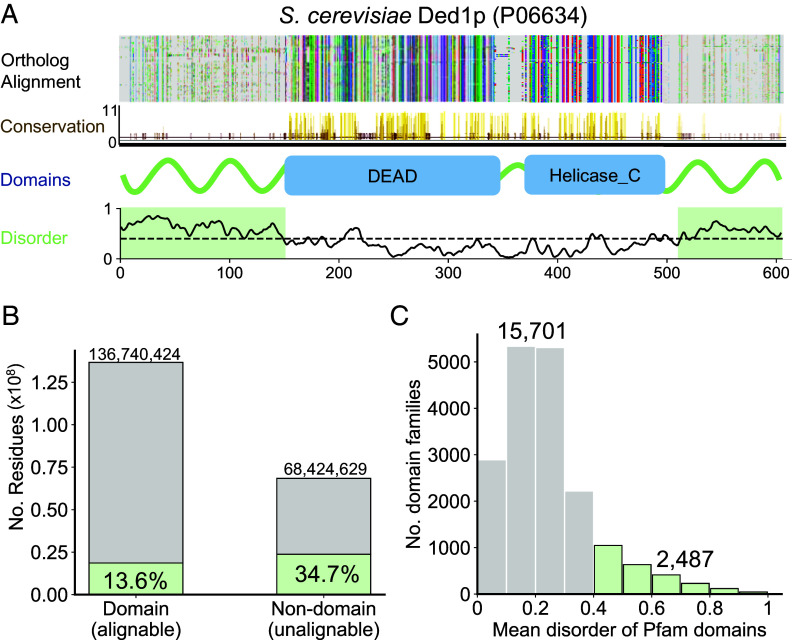
IDRs are common in the proteome and difficult to align. (*A*) Multiple sequence alignment of Ded1p orthologs. The disordered N- and C-terminals (shaded in green) contain a large number of gaps (gray) in the alignment and lack domain annotation due to poor alignment and conservation of amino acid physicochemical properties (as defined by JalView, *Materials and Methods*). On the contrary, Pfam domains (blue boxes) correspond to well-aligned, ordered regions with conserved sequences. (*B*) Of all ~205 million residues in UniProt, ⅓ do not belong to Pfam domains, representing unalignable residues without systematic functional annotation. Disordered residues are enriched in non-domain (unalignable) regions with greater than twofold relative enrichment compared to domains. (*C*) Disorder is poorly represented in the Pfam database (release 34.0). Of the seed sequences used to construct each domain-specific profile HMM of 18,188 domain families analyzed, only 2,487 families (14%) contain seed sequences with at least 0.4 mean disorder (*Materials and Methods*).

Alignment-based algorithms are also unsuitable for most intrinsically disordered regions (IDRs) (12–14). These are flexible sequences that evolve more rapidly than ordered domains since they lack constraints to form a stable 3D structure (15–18). As a result, they harbor more substitutions and insertions/deletions (InDels) between homologs than ordered sequences (19, 20). As illustrated with the multiple sequence alignment of *Saccharomyces cerevisiae* Ded1p orthologs (Fig. 1*A*), the N- and C-terminal IDRs of Ded1p are poorly aligned, indicated by the large fraction of gaps and amino acid mismatches. Additionally, IDRs often contain biased amino acid compositions and repetitive regions which are not amenable to alignment-based algorithms (21, 22). Consequently, IDRs are largely ignored when identifying homologous sequences, where sensitivity is mostly derived from alignment of conserved domains.

Whereas IDRs have previously been thought of simply as flexible linkers between the functional structured domains, rapidly increasing evidence has demonstrated the importance of IDRs in modulating protein function (18, 23). Due to their inherent flexibility, multivalency, and ability to sample multiple conformations, they mediate a wide variety of functions including molecular recognition, protein modification, molecular assembly (18), and biomolecular condensate formation (24–29). For example, the IDRs of Ded1p play an integral role in regulating its condensation and translation repression (30). Despite ample evidence of IDRs as functional regions under conservation, homology assessment of IDRs remains a bottleneck.

The underlying principles of alignment require a collinear correspondence between residues and assume that the two sequences can be connected via a mutational path of substitutions, insertions, and deletions (31). However, beyond a certain level of divergence, this approach becomes inaccurate. In rapidly evolving IDRs, primary sequence conservation is generally lower because only shorter functional regions, such as SLiMs, repeat regions, hydrophobic patches, or in certain cases amino acid compositional characteristics may be conserved across orthologs. These conserved features in disordered regions are essential for IDR/protein function, (32–38) but would often be overlooked using alignment methods (13). Recent work by Singleton and Eisen has shown extensive evidence of constrained evolution in the IDRs of *Drosophila* (38), corroborating earlier findings of conservation in IDRs of *S. cerevisiae* (14) and highlighting that conservation of such features is common across multiple organisms. However, these feature-based analyses are susceptible to inherent biases in feature selection and the nonindependence between individual features. Accordingly, besides the development of an alignment-free strategy to assess homology in such regions, a more comprehensive consideration of all these features—not only amino acid composition but also of longer sequence regions which may confer specific binding/ physicochemical characteristics, is required for accurate and sensitive detection of homologs.

Here, we developed SHARK (Similarity/Homology Assessment by Relating K-mers), an alignment-free algorithm that compares the overall amino acid composition and short motifs shared between sequences. We trained SHARK-dive, a homology classifier on a curated set of unalignable homologous sequences enriched in disordered regions. On these sequences, SHARK-dive outperforms conventional alignment methods [SW (6), BLAST (7), and HMMER (9)] in detecting evolutionarily related homologs. Furthermore, SHARK-dive distinguishes functional IDR homologs (i.e., analogs) from functionally unrelated sequences reported in the literature. SHARK-dive not only offers predictions of homologous IDRs but also highlights the sequence properties and regions that may contribute to homology. Our tools can be used to facilitate systematic assessment of homology in difficult-to-align sequences and generate hypotheses of sequence–function relationships of unalignable, disordered sequences.

## Results

### Unalignability and Disorder are Common in the Protein Universe.

In order to systematically examine the limitations of sequence alignment, we assessed the prevalence of disordered and unalignable regions across UniProt sequences. Using the disorder predictor IUPred2A (39–41), we found that 21% of the ~205 million residues in SwissProt were predicted to be disordered (Fig. 1*B*) and over 60% of proteins contain an IDR at least 10 amino acids long (*SI Appendix*, Fig. S1), indicating that disorder is pervasive. To quantify the degree to which sequences are alignable, we defined unalignable sequences as difficult-to-align regions which do not map to domains according to Pfam (10), and will use the term “unalignable” as a synonym for “difficult-to-align” throughout. While this is nonetheless a simplified prediction, we found that a third of all residues still lack Pfam annotation and would be considered unalignable despite extensive, continuous curation of Pfam (Fig. 1*B*). At the protein level, this translates to ~5% of proteins in UniProt (*SI Appendix*, Fig. S2) lacking a Pfam annotation, with varying degrees of Pfam coverage for different organisms (~8 to 29%, *SI Appendix*, Fig. S3).

To identify relationships between disorder and alignability, we annotated every residue as either ordered /disordered (based on its IUPred score) and aligned/unalignable (based on its inclusion in a Pfam domain). Whereas only 14% of aligned domain residues are disordered, a far higher fraction (35%) of unalignable residues are disordered (Fig. 1*B*). This is corroborated by a similar analysis performed using the state-of-the-art disorder predictor flDPnn (42, 43) (*SI Appendix*, Fig. S4). This analysis also revealed a significant fraction of ordered residues which were not mapped to domains, implying that even some structured residues are not amenable to alignment for homology assessment and functional annotation. The poor alignability of IDRs is further highlighted by the low number of disordered seed sequences used to build Pfam families (Fig. 1*C*). Altogether, this indicates that databases such as Pfam cannot completely categorize and functionally annotate sequences, particularly IDRs, which we reasoned is due to the limitations of alignment.

### SHARK-scores are Alignment-free, Physicochemistry-encoded Parameters to Compare Sequences.

**Fig. 2.**
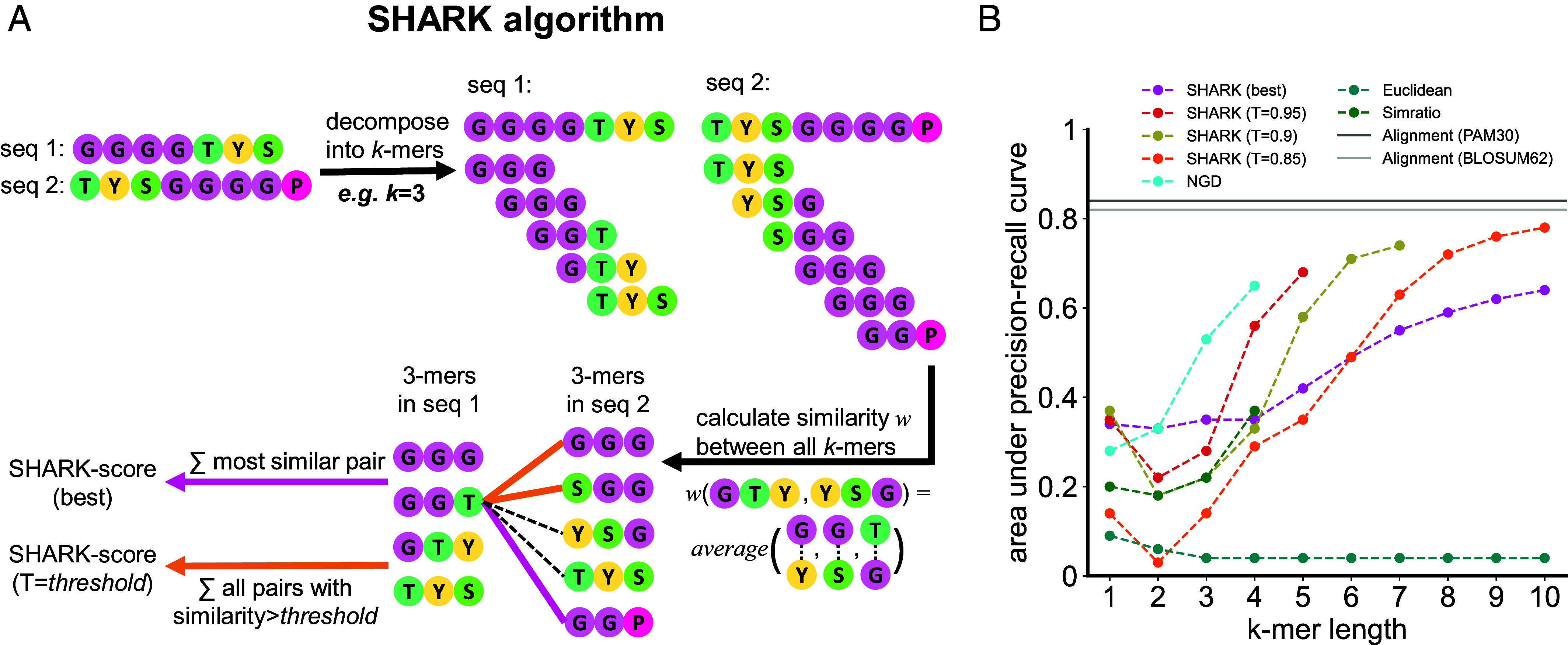
Overview of the SHARK algorithm and SHARK-scores. (*A*) SHARK assesses sequence similarity by representing each sequence as subsequences of length *k* (*k*-mers) and then assessing the k-mer similarities between the sequences. Either the most similar *k*-mer is chosen to give the SHARK-score (best) variant or all *k*-mers with similarity (*w*) greater than a threshold (T) are summed to give the SHARK-score (T). Unlike most alignment-free scores where a low value indicates higher similarity, a high SHARK-score between sequences indicates high similarity. (*B*) Homology detection performance on the alignable-disorder dataset. SHARK outperforms existing alignment-free metrics in homology assessment performance, especially with longer *k*-mers where existing metrics lose performance. SHARK-scores achieve auPRC values greater than 0.65, which is the highest performance attained by existing alignment-free methods (Normalized Google Distance, NGD, at *k* = 4). For each algorithm, only *k*-mer lengths where the auPRC would not be overestimated due to the lack of similar/identical *k*-mers between homologs were plotted (*Materials and Methods*, *SI Appendix*, Fig. S7). Nonetheless, local alignment still offers superior performance (see *SI Appendix*, Table S1 for details on parameters used).

In order to overcome the fundamental constraints of alignment, Similarity/Homology Assessment by Relating K-mers (SHARK) was developed to compare sequences in an alignment-free manner (Fig. 2*A*). Consistent with existing word-based approaches, SHARK first decomposes each sequence into overlapping subsequences of a particular length *k*, which we refer to as *k*-mers (also known as *l*-tuples/*n*-grams/words). Whereas *k*-mer-based approaches have been long-established (44–46), they only recognize identical *k*-mers. Here, we enable the comparison of nonidentical but sufficiently similar *k*-mers by assessing their physicochemical properties according to the Grantham distance matrix (47) which considers side-chain chemical composition, polarity, and size. A *k*-mer similarity matrix is then constructed by comparing all *k*-mer pairs and aggregated into two score variants representing the similarity between the sequences: SHARK-score (best) considers only the most similar *k*-mer, whereas the SHARK-score (*T*) variant considers all similar *k*-mers above a threshold *T* (*Materials and Methods*).

To evaluate the efficacy of SHARK-scores in homology assessment, an initial proof-of-concept benchmark revealed that SHARK-scores were superior to local alignment as well as several alignment-free metrics at distinguishing between the functionally opposing N- and C-terminal IDRs of Ded1p orthologs (*SI Appendix*, Fig. S5). More importantly, on a comprehensive set of 143 Pfam protein families with the highest overall disorder content referred to as the alignable-disorder dataset (*SI Appendix*, Fig. S6 and Dataset S1), SHARK-scores outperformed existing alignment-free metrics in identifying homologous domain sequences in terms of highest auPRC attained, afforded by consistently superior performance for longer *k*-mers (Fig. 2*B*). While SW local alignment was still superior (Fig. 2*B* and *SI Appendix*, Table S1), the performance improvements of SHARK-scores suggest that they may complement existing alignment-free algorithms, particularly at longer *k*-mer lengths where existing methods consistently struggled.

### SHARK-dive Is a Simple Machine learning Model Trained on a Set of Unalignable, Orthologous Sequences.

Despite the improvements of SHARK-scores over existing alignment-free metrics in IDR homology assessment, alignment-free approaches were inferior to alignment in the alignable-disorder dataset. We reasoned that since the ground-truth of homology for the dataset is ultimately defined from alignment-based HMMs, this inherently advantaged local alignment in homology assessment. Accordingly, instead of benchmarking against alignable sequences, we aimed to investigate the performance on homologous but unalignable sequences.

**Fig. 3.**
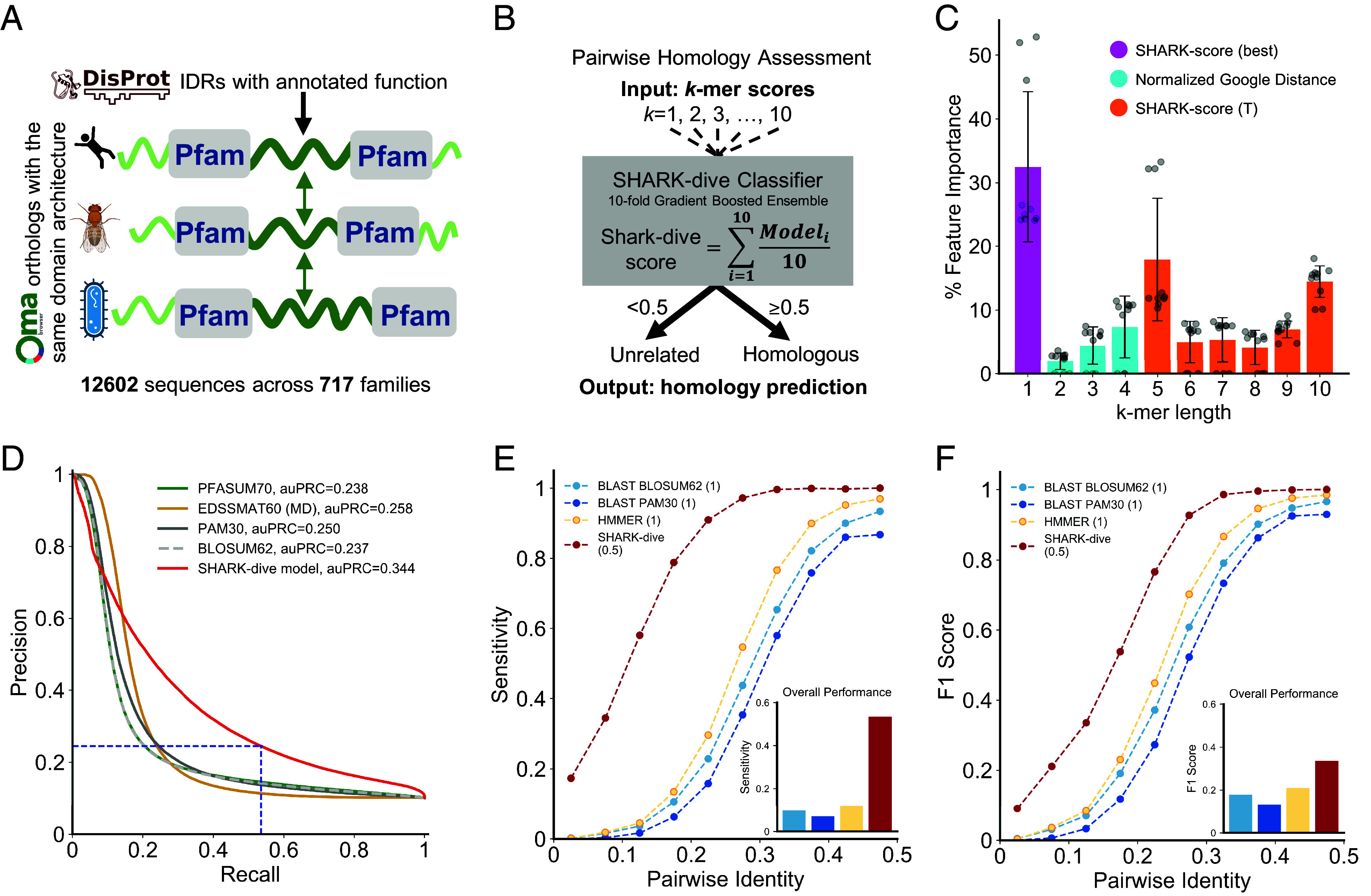
SHARK-dive outperforms conventional alignment in assessing homology in unalignable, disordered sequences. (*A*) Curation of the unalignable-ortholog dataset. We collected sequence regions that are flanked by the same domains in sequences with identical domain architectures, with functional annotation (from DisProt) for at least one representative ortholog (orthogroups from OMA). Following filtering for max. 50% identity, we curated 12,602 unalignable and highly disordered sequences across 717 sequence families. (*B*) SHARK-dive is an ensemble gradient-boosting classifier of 10 submodels trained on a training subset of families of the unalignable-ortholog dataset using 10 *k*-mer scores as input. (*C*) Feature importance of SHARK-dive. Each bar represents the mean feature importance of the scores for each value of *k* across the submodels (error bars indicate SD). Magenta and orange bars represent SHARK-scores (best and T variant, respectively), and cyan bars represent Normalized Google Distance used for *k*=2 to 4. Gray circles show the feature importance of individual submodels (*D*) Precision*–*Recall performance of SHARK-dive compared with alignment with various substitution matrices on the unalignable-ortholog test sequences. For the EDSSMAT series of matrices, only the best-performing one is plotted. Dashed blue lines refer to the performance at 0.5, the default threshold chosen, where SHARK-dive achieves far higher recall at the same level of precision. (*E*) Among the homology detection tools tested, SHARK-dive offers the highest sensitivity to remote homologs across multiple pairwise sequence identity (PID) bins. Overall, SHARK-dive offers over fourfold improvement over conventional alignment-based tools BLAST and HMMER (at an E-value threshold of 1, thresholds shown in brackets). Shown in the inset is the overall sensitivity across all PID bins. (*F*) SHARK-dive offers superior remote homology detection performance over conventional alignment-based methods, particularly when sequence pairs share very low identity. Shown in the inset is the overall F1 performance across all PID bins. Thresholds used for each method are shown in brackets. Performance for a more lenient E-value threshold of 10 is shown in *SI Appendix*, Fig. S14.

However, a large-scale reliable ground-truth dataset was lacking. We therefore adopted an evolutionary-based approach, first using the DisProt database (48) to identify proteins that contained IDRs with annotated functions which are likely under selection to conserve functional features/motifs, then finding their orthologs using the OMA orthology database (Fig. 3*A*) (49). This allowed correspondence to be assigned between nondomain segments for orthologs with an identical domain architecture solely based on their surrounding domains, without performing sequence alignments (Fig. 3*A*). These sets of segments (henceforth referred to as a “sequence family”) were aggregated to form the unalignable-ortholog dataset (Dataset S2). We note that although there is no explicit disorder requirement for the orthologous sequences, the majority (70%) of these sequences were indeed disordered (*SI Appendix*, Figs. S8–S10), substantiating the difficulty in aligning IDRs. In accordance with typical machine learning protocols, the unalignable-ortholog dataset was split by sequence family for model training, validation, and testing (*SI Appendix*, Fig. S9 and Dataset S2).

SHARK-dive is a machine-learned homology classifier trained on the unalignable-ortholog training dataset. To integrate information and homology assessment power across *k*-mer lengths, we chose the best algorithm for each *k*-mer length from *k* = 1 to *k* = 10, in accordance with the typical motif lengths in IDRs (50, 51), based on performance on the alignable-disorder dataset (*SI Appendix*, Table S2). Architecturally, SHARK-dive is a simple 10-fold ensemble of gradient-boosted decision tree classifiers requiring only the 10 *k-*mer scores as input (Fig. 3*B* and *Materials and Methods*). Upon training, we observed that while each *k*-mer length contributed to model decision-making, *k* = 1, 5 and 10 were consistently the most important features (Fig. 3*C*). Since 5- and 10-amino acid-long peptides correspond closely to 1 to 2 helical turns (52), we used Agadir (53) to identify whether helix-formation tendency underlay the importance of *k* = 5 and 10, but were unable to detect significant helix propensity among the sequences (*SI Appendix*, Fig. S11).

### SHARK-dive has Superior Sensitivity to Alignment in Detecting Unalignable, Disordered Homologs.

To quantify the performance of SHARK-dive, we assessed its ability to detect homology from an all-vs-all comparison of unalignable-ortholog test sequences that were withheld from model training. SHARK-dive offered superior overall performance and outperformed SW local alignment across all substitution matrices tested (*SI Appendix*, Table S3), including the PFASUM70 matrix (54) which had been shown to be the best-performing set of parameters for an alignment task based on Pfam-A domains (55), as well as the EDSSMAT series of disorder-optimized substitution matrices (56) (Fig. 3*D* and *SI Appendix*, Table S1). We further assessed the threshold-specific performance of SHARK-dive using the default classifier threshold of 0.5 (the discriminatory power of this threshold is shown in *SI Appendix*, Fig. S12). SHARK-dive achieved a 33% increase over the highest F1 score attained by alignment [EDSSMAT60 (MD)], along with a 32% increase over the most sensitive alignment (PFASUM70, *SI Appendix*, Table S3). We found this surprising since alignment thresholds were defined by the maximum F1 score attained (*SI Appendix*, Table S3), which gives it an inherent advantage.

SHARK-dive was also benchmarked against the most widely used homology search tools, BLASTp (BLAST) and pHMMER (HMMER). Using default search parameters and reporting thresholds (E-value ≤ 1 and 10, *SI Appendix*, Table S4), these tools were incapable of reporting even 25% of homologs (*SI Appendix*, Table S5). In contrast, SHARK-dive achieved the highest F1 performance across all tools and parameters tested (Fig. 3*F* and *SI Appendix*, Table S5), largely by surpassing the sensitivity bottleneck with a vast increase in the number of true homologs detected (Fig. 3*E*, *Inset*), including a large fraction which is undetectable by BLAST/HMMER (*SI Appendix*, Fig. S13).

### SHARK-dive Identifies Unalignable yet Evolutionarily Homologous as well as Functionally Analogous IDRs.

**Fig. 4.**
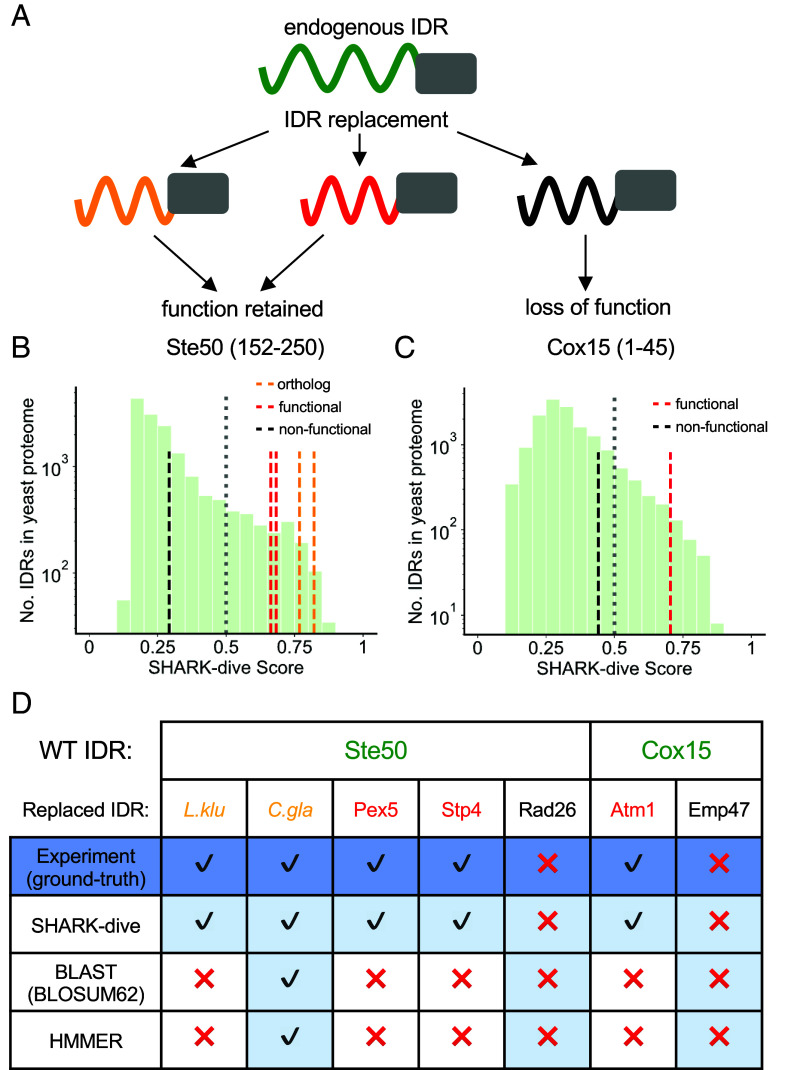
SHARK-dive accurately identifies functional IDR homologs reported in the literature. (*A*) Schematic of IDR replacement experiments, where the endogenous IDR (green) is replaced by different IDRs. Some IDRs, including IDRs in orthologs, can rescue protein function (red and orange, respectively), whereas others cannot (black). (*B*) Highly dissimilar IDRs can rescue Ste50 function upon replacement of its endogenous IDR. IDRs in Ste50 yeast orthologs (orange lines) were able to confer Ste50 WT-fitness and were also predicted as homologous (57). Pex5 and Stp4 IDRs capable of functional rescue (red lines) were predicted to be homologous to Ste50 IDR, whereas the loss-of-function-inducing Rad26 IDR was not (14). Further, SHARK-dive predicts other IDRs in the yeast proteome (green bars) that may also be homologous to the Ste50 IDR (IDRs above the SHARK-dive threshold, gray line). (*C*) Cox15 localization can be rescued upon replacement of its endogenous IDR by Atm1 IDR. SHARK-dive correctly predicts Atm1 IDR to be homologous, while the Emp47 IDR was not (14). Similarly, other IDRs in the proteome are also predicted to be similar. (*D*) Summary of prediction accuracy by SHARK-dive and other homology assessment tools on Ste50 and Cox15 IDR homologs, as determined by experimental results. Light blue cells indicate homology predictions consistent with experimental results (dark blue).

To further validate SHARK-dive, we investigated its ability to identify functionally homologous IDRs reported in the literature. Specifically, we searched for functional rescue experiments, where protein function was assayed following the replacement of an endogenous IDR (Fig. 4*A*). The replaced IDRs share low sequence identity (*SI Appendix*, Table S6) and align poorly with the endogenous IDR (*SI Appendix*, Fig. S15). We then assessed whether SHARK-dive and existing tools can identify IDRs capable of functional rescue as being homologous. Importantly, we have ascertained that the sequences were not used for model training.

Recent work by Zarin *et al.* investigated the effect of replacing the IDR of *S. cerevisiae* Ste50, a mating pathway adaptor (57). Replacing the endogenous IDR with evolutionarily related orthologous IDRs from other yeast species was sufficient to maintain WT-like fitness (57). SHARK-dive not only correctly identified the homology relationship for orthologous IDRs (Fig. 4*B*, orange lines) but was able to distinguish *S. cerevisiae* IDR analogs capable of functional rescue (Pex5 and Stp4, red lines) from the Rad26 IDR which was incapable of functional rescue (black line) (14). Contrastingly, both HMMER and BLAST failed to detect all homologs and analogs; both tools detected only the *Candida glabrata* homolog below the 0.05 threshold (*Materials and Methods*), and BLAST could not detect analogy to the Stp4 IDR even with a high E-value threshold of 10.

Importantly, the individual *k-*mer scores, i.e., inputs to the SHARK-dive model, highlight the shared homology, where the orthologous IDRs and the functional analogs share generally higher *k-*mer scores not only compared to the nonfunctionally replacing Rad26 (*SI Appendix*, Fig. S16*J*) IDR but also to the distribution of unrelated sequences (*SI Appendix*, Fig. S16 *B*, *D*, *F*, and *H*). Moreover, since the basal net charge of the Ste50 IDR has been reported to be important in conferring functional output, it is interesting that all the reported functional and evolutionary homologs have a high amino acid composition (*k =* 1) score and similar amino acid compositions, particularly in terms of charged residues glutamic acid, aspartic acid, lysine, and arginine (*SI Appendix*, Fig. S16 *A*, *C*, *E*, and *G* vs. *I*, boxed in red). This suggests that the individual *k-*mer scores can contain useful insights that underlie the prediction of homology.

In another set of experiments, mitochondrial localization was restored upon replacing the N-terminal IDR of *S. cerevisiae* cytochrome oxidase Cox15 with the Atm1 IDR (14). This functional homology was correctly predicted by SHARK-dive, whereas the Emp47 IDR, which could not restore mitochondrial localization, was correctly predicted not to be homologous (Fig. 4*C*). Neither BLAST nor HMMER could detect Cox15-Atm1 IDR homology (Fig. 4*D*). Interestingly, SHARK-dive was also capable of identifying synthetic IDRs, designed to share similar molecular/biochemical properties as Cox15 IDR, and also capable of conferring mitochondrial localization upon replacement (58) (*SI Appendix*, Table S7). Altogether, SHARK-dive accurately predicted functional homology of IDRs across different functions/phenotypes despite negligible sequence similarity. Likewise, insights can be gleaned from the *k-*mer scores and matches in the Cox15 IDR swap experiments, where the Atm1 IDR harbors 10-mer regions that are similar to the Cox15 IDR (*SI Appendix*, Fig. S17 *A*–*C*). These regions (e.g., PKVKTQVKKT) contain many basic residues, which are known to be properties of canonical mitochondrial localization signals (59). These similar regions are notably absent from the Emp47 IDR (*SI Appendix*, Fig. S17 *D*–*F*), which may underlie its inability to confer mitochondrial localization.

### SHARK-dive Offers Interpretable Predictions of Evolutionary Homologs and Functional Analogs.

**Fig. 5.**
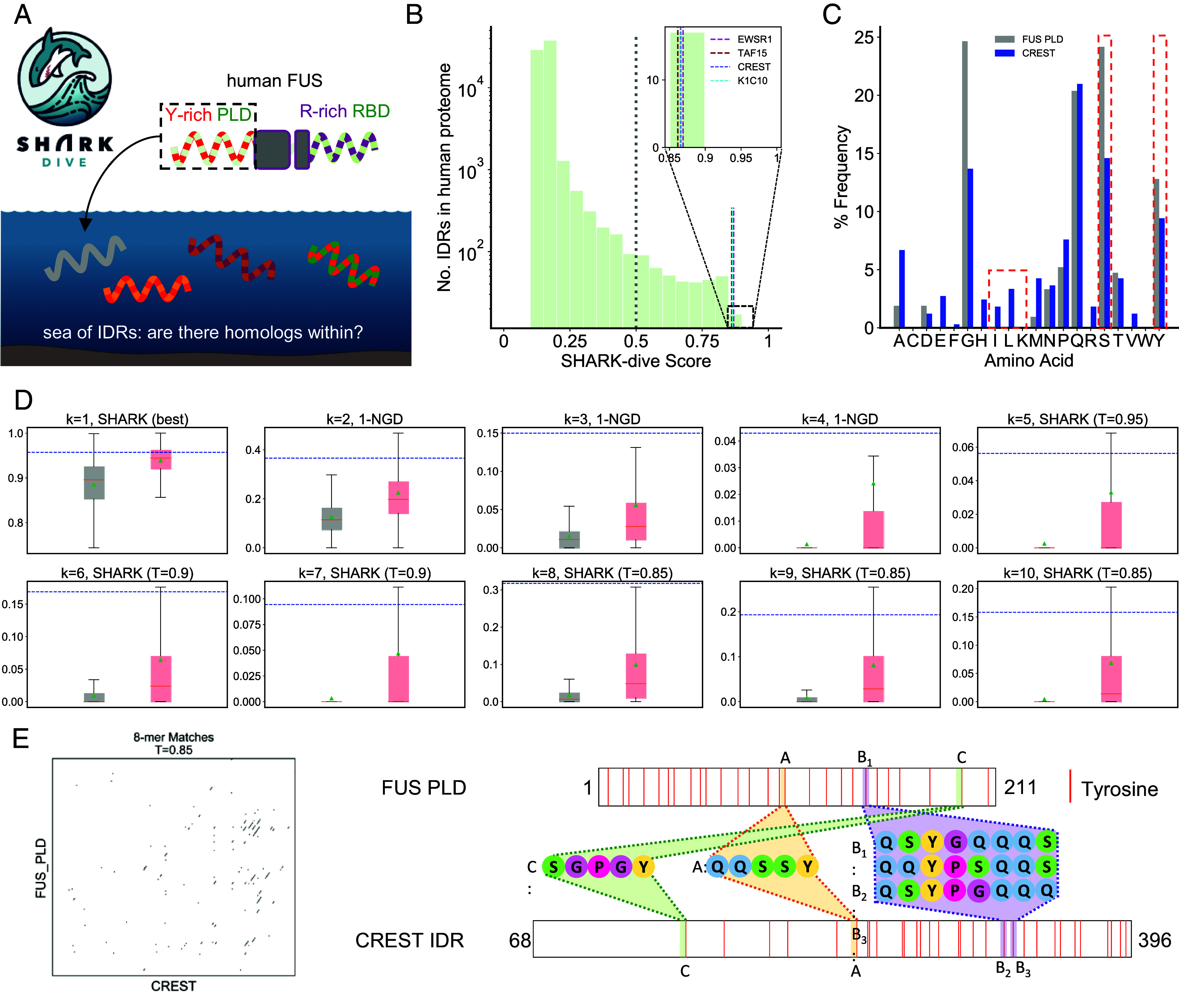
SHARK-dive offers interpretable predictions of homologous IDRs. (*A*) Schematic of the homology search with human FUS PLD (residues 1 to 211 as defined in ref. 32) to find similar IDRs. (*B*) SHARK-dive predicts various IDRs in the human proteome to be homologous, including N-terminal IDRs of known FET family members TAF15 and EWSR1, as well as a variety of unrelated IDRs in proteins such as transcriptional activator CREST and Type 1 Keratin (K1C10). (*C*) Human CREST IDR and FUS PLD share similar amino acid compositions, particularly in their high tyrosine and serine contents and low abundance of lysine and aliphatic amino acids (highlighted in red). (*D*) To interpret the homology prediction of SHARK-dive, the input features (*k*-mer scores) can be compared to the distribution of unrelated sequences or true homologs in the training sequences to identify particular *k*-mer lengths which may contribute to prediction of homology (or lack thereof). In the case of the CREST IDR which is confidently predicted to be homologous, all *k*-mer scores are higher than the average homologous pair. For *k* = 2 to 4, NGD scores are visualized as 1-NGD (otherwise known as the Normalized Google Similarity) such that higher scores represent higher similarity for ease of interpretation. Boxplots show median (orange line), mean (green triangle), and quartiles. Outliers are not shown but can be found in *SI Appendix*, Fig. S23. (*E*) The inputs to SHARK-dive are similarity-matrix derived *k*-mer scores, which can be visualized to show regions of high similarity between sequences. This can be visualized for a range of *k*-mer lengths as well as similarity thresholds (*k* = 8 and T = 0.85 shown here). Following this, similar *k*-mers identified from the similarity matrix can be mapped back onto the sequence to identify potential motifs or sequence characteristics that may contribute to shared function/homology (3 examples here shown). Potential motifs can be in different positions along the sequence such as motifs *A* and *C*. The similarity matrix also allows similar but non-identical motifs to be identified (e.g., B_1,2,3_) as well as detection of multiple motifs (e.g., B_2_ and B_3_ are both similar to B_1_). Inspection of the shared motifs suggests that besides the tyrosine and serine residues, glutamine blocks (e.g., QQ) may also contribute to shared homology between CREST IDR and FUS PLD.

Given SHARK-dive’s accuracy in identifying experimentally verified functional IDR homologs, we used SHARK-dive to generate predictions of homologous IDRs. We used the human FUS, which has been shown to form in vitro condensates (32) and is recruited to stress granules in vivo (60, 61), as an example. Specifically, the disordered N-terminal prion-like disordered region of human FUS (FUS PLD, residues 1 to 211, Fig. 5*A*) mediates condensate formation via homotypic Tyr–Tyr π–π interactions (62) or Tyr–Arg π–π and Arg–Tyr cation–π interactions with its arginine-rich RNA binding domain (FUS RBD, residues 212 to 526, Fig. 5*A*) (32). We therefore hypothesize that sequences homologous to the FUS PLD may be able to interact with FUS or perform a similar functional role in driving phase separation and biomolecular condensate formation.

Therefore, we used SHARK-dive to perform a blind search of the FUS PLD against a set of IDRs extracted from the proteomes of human and 6 other common model organisms (*Materials and Methods* and *SI Appendix*, Table S8) as an example usage of SHARK-dive for homology searches. SHARK-dive not only predicted homology to the N-terminal IDR of the mouse and zebrafish FUS orthologs (as the top hit, Dataset S3) but also to the PLD-containing N-terminal IDRs of FET-family members (63) TAF-15 and EWSR1 that are known to phase separate (32) (Fig. 5*B*).

As an example, SHARK-dive predicted an IDR in the human CREST protein to be homologous (Fig. 5*B*). CREST is a highly disordered transcription activator and regulator of cortical neuron dendritic growth (64, 65). Similar to FUS, it is also implicated in amyotrophic lateral sclerosis (66, 67). It contains a C-terminal IDR that shares only 15% identity with FUS PLD, and is not reported in a BLAST search of the FUS PLD (from NCBI BLAST webserver). The predicted homology arises not only from a similar amino acid composition (Fig. 5*C*) but also due to the presence of multiple similar *k*-mer patterns (*SI Appendix*, Fig. S18), which give a respective *k*-mer score which is higher than most unrelated sequences (Fig. 5*D*). We further note that some 5-mers are swapped in order between the two sequences and thus would be difficult for alignment to fully detect (Fig. 5*E*). Moreover, homology was not predicted between FUS RBD and the CREST IDR, indicating that the prediction is specific to the IDR, and not due to the full-length protein.

As a control, we report that a predicted non-homolog, ZN365, shows markedly fewer *k*-mer matches and lower scores similar to those of unrelated sequences used to train SHARK-dive (*SI Appendix*, Fig. S19*A*). Since both FUS (68) and ZN365 (69) have also been shown to be involved in double-stranded DNA break repair, suggesting that functional similarity of the protein overall is independent of the IDR homology prediction and that SHARK-dive can be used to predict IDR homology independent of the function of the full-length protein.

To investigate whether SHARK-dive predictions from the blind sequence-database search could be biologically relevant, we decided to conduct a brief literature search of some interesting top-ranking candidates to provide support for the validity of SHARK-dive predictions. Compellingly, literature review revealed indirect experimental support for CREST IDR’s functional homology to the FUS PLD, as CREST has been reported to be capable of phase-separation and can even interact with the FUS PLD (66, 67).

Highlighting the ability of SHARK-dive to identify possible homologous IDRs despite a lack of shared protein function, FUS PLD was also predicted to be homologous to the C-terminal IDR of human Type I Keratin (K1C10/Krt10), which shares no functional similarity to FUS at all; it is involved in skin epidermal barrier formation (70). Despite this, corresponding *k*-mers can be found between FUS and the keratin C-terminal IDR (*SI Appendix*, Fig. S19*B*). Fascinatingly, studies have evidenced the ability of K1C10 to interact with phase-separated, liquid-like keratohyalin granules (KGs) (70); Quiroz et al. further demonstrated that the N- and C-terminal low complexity (LC) domains of Type 1 keratin are critical in driving this interaction, as LC domain tagged mCherry is capable of partitioning into KG condensates. We chose the keratin example to highlight that proteins without direct functional similarity to each other may share IDRs that contribute to a particular phenotype, in this case phase separation.

Finally, we highlight the RALY IDR as an example where a low compositional similarity is overcome by the presence of similar *k*-mer matches to give a prediction of homology (*SI Appendix*, Fig. S20). Interestingly, FUS and RALY are components of the same ribonucleoprotein complexes (RNPs) (71), which provides a possible biological rationale for their predicted homology since IDRs are important in promoting RNP formation and can encode RNP identity (72).

Surprisingly, Gasperini *et al*. identified an interaction between FUS and RALY required for RNP formation, and follow-up domain deletion experiments revealed loss of interaction between a C-terminal truncation (Δ360-501 FUS-GFP) and RALY, indicating that the RGG rich RNA binding domain (FUS RBD) was involved in FUS–RALY interactions. Surprisingly, SHARK-dive also predicted homology between the FUS RBD and the RALY IDR (*SI Appendix*, Fig. S21). Accordingly, the proposed role of cation-π and π-π interactions in driving protein interaction and phase separation, particularly in the FUS IDRs (32, 62), provides some support to the FUS RBD-RALY homology prediction.

In sum, we showed an example of how from a blind sequence-database search (akin to a BLAST search), SHARK-dive can predict homology relationships that may be biologically/functionally relevant. Since none of the IDRs were used in training SHARK-dive, we believe that these homology predictions, combined with reported experimental evidence of functional similarities, suggests that SHARK-dive can predict evolutionary homology and functional analogy even if the sequences share low sequence identity. We further note that besides the human proteome, SHARK-dive identified several IDRs in other organisms to be similar to the FUS PLD (an example in yeast is shown in *SI Appendix*, Fig. S22, all results are available at Dataset S3). Coupled with the ability to determine the regions that contribute to the predicted similarity between sequences, we believe SHARK-dive offers the ability to generate hypotheses regarding IDR homology and function across proteomes and species.

## Discussion

Alignment-based homology detection has exponentiated our understanding of sequence–function relationships and aided functional annotation in structured regions but has had limited benefit for disordered and rapidly evolving regions. Here, we present the SHARK algorithm and its derivatives SHARK-scores and SHARK-dive, developed specifically to tackle this challenge. It is based on an alignment-free, *k*-mer-based approach which breaks free from the inherent constraints and limitations of sequence alignment, and innovates by assessing the similarity between *k*-mers in physicochemical space. K-mer-based methods were successfully applied to several problems such as comparing genomes with low sequence coverage (73) and large sequencing data (74), and phylogeny reconstruction (75). Here, we show how a simple *k-*mer-based method can be applied to compare protein sequences with very low sequence identity. The SHARK algorithm amalgamates the strengths of both alignment and alignment-free approaches by combining the removal of collinearity constraints with the consideration of *k*-mer similarity.

Despite the improvement of individual SHARK-scores over existing alignment-free algorithms, we reasoned that accurate homology detection for disordered, unalignable regions requires consideration of amino acid composition as well as *k*-mers of varying lengths. Besides the performance gain in detecting remote homologs, this was also biologically motivated since functional motifs may be of varying sizes. We therefore harnessed the power of machine learning in aggregating a range of *k-*mer scores to train the SHARK-dive model. In the future, it would be interesting to extend SHARK to compare multiple sequences and have an iterative search approach of large databases analogous to alignment-based search algorithms such as PSI-BLAST (7) and JackHMMER (76) to further increase sensitivity to remote homologs.

Since previous remote homology assessment benchmarking datasets were based on ordered/structured regions (e.g., SCOPe, ASTRAL) (46) or alignable sequences (e.g., Pfam) (55), here, we curated a dataset of unalignable orthologous by identifying nondomain sequences between known orthologs that share identical domain architectures and are flanked by the same set of domains (Fig. 3*A*). Our domain-based approach is an alignment-independent extension of the protocol used by Lu *et al.* and similar to approaches adopted in experimental contexts (57, 77, 78). Indeed, the reverse-homology-based tool by Lu et al. (77) together with SHARK-dive showcases the potential of moving beyond sequence alignments: where the former adopts this rationale to assume homology and thereby identify conserved motifs/regions within, SHARK-dive attempts to further discover homology. There are nonetheless limitations of our approach, such as the assumption of nonhomology between different sequence families, which is difficult to guarantee without extensive experimental evidence. This is nonetheless a general assumption held also by existing benchmarks, which we aimed to alleviate with the strict identity filtering (<50% identity) applied to our sequences (*Materials and Methods*).

Importantly, while the unalignable-ortholog dataset is enriched in IDRs (*SI Appendix*, Figs. S8–S10), it makes no explicit requirement for the sequences to be disordered. Instead, it assumes that the functionally annotated IDRs in the DisProt database (48) preserve their functions across evolution and therefore yield signatures of conservation and homology among orthologs, which SHARK-dive aims to detect. Accordingly, this approach is inspired by the tenets of evolutionary biology, where functional regions/properties are conserved across evolution, which forms the basis of sequence homology-based annotation transfer (3–5). By training SHARK-dive on this set of orthologs, we hypothesize that the model could also identify functional homologs that do not share a common ancestor, as demonstrated in SHARK-dive’s unique versatility in identifying both evolutionarily related IDRs from orthologs as well as IDR analogs without including false positives (Fig. 4).

Curation of the unalignable-ortholog dataset also enabled a systematic analysis of the suitability of existing alignment tools in detecting relationships in these difficult-to-align sequences. We report that SHARK-dive outperforms Smith-Waterman local alignment in terms of overall performance (auPRC, Fig. 3*D*). While local alignment achieved higher precision in the high-score, low sensitivity range (*Left* part of the PR curve), this is unsurprising because these represent the most similar (including identical) sequences where alignment would be the optimal algorithm. Instead, SHARK-dive outperformed local alignment across multiple substitution matrices and gap parameters (*SI Appendix*, Table S3) and particularly in the high sensitivity range (*Right* part of the PR curve) where the most dissimilar homologs exist, and where sequence alignment is weakest at remote homology detection. This is substantiated by the low sensitivity of widely used alignment-based homology detection tools such as BLAST and HMMER (only ~20% homologs detected, *SI Appendix*, Table S5), even with continued improvements (79, 80). Taken together, this indicates a significant bottleneck in their sensitivity to remote unalignable homologs. Here, SHARK-dive can fill an unmet need in providing accurate predictions of remote homology (as assessed by F1 score, *SI Appendix*, Table S5) with significantly higher sensitivity. We believe that, despite the corresponding trade-off in precision, the increased sensitivity provides a substantial net benefit in allowing experimentalists to look beyond the most similar homologs.

With the recent advances in deep learning and natural language processing, methods such as DeepBLAST (81), ProtENN (82), DEDAL (55), and pLM-BLAST (83) revisited the pairwise alignment algorithms developed in the 1980–90s. These models harnessed large amounts of sequence data and computing power to improve on alignment accuracy by optimizing parameters such as substitution scores and gap penalties. While they showed improvements in the detection and alignment of homologous sequence pairs with low sequence identity, they assume that an accurate alignment exists and assessed performance primarily on ordered domains (55, 81, 82). In contrast, our method was trained on nondomain, disordered regions, and it is a relatively simple machine learning model based on interpretable features that directly translate into sequence motifs that can be shared between sequences without the need for linear correspondence between sequence positions. Since language models use the corpus of UniProt to train, it is difficult to create an unbiased set of sequences from our unalignable orthologs dataset. Therefore, we evaluated the performance on the experimentally studied sequences and compared SHARK to DEDAL which we consider the state-of-the art deep learning model for improved SW alignment (*SI Appendix*, Fig. S24 and Table S7). While DEDAL outperforms HMMER and BLAST, it misses several true positives that only SHARK-dive could identify.

This increased sensitivity of SHARK-dive may be especially useful when BLAST and HMMER do not offer any interesting or reasonable predictions to a wide variety of cases ranging from IDRs, to structured regions, and even to whole proteins (*SI Appendix*, Fig. S25 for results on a subset of domain-less proteins in the unalignable-orthologs test dataset). In practice, SHARK-dive can be used in the same way as alignment-based homology search tools, where one or more query sequences can be searched against a list of targets in a sequence database to give aquery-versus-target SHARK-dive homology prediction score. The SHARK-dive classifier score represents the likelihood of the two sequences being homologous where scores ≥0.5 represent a positive prediction of homology (evolutionary or functional). We note that the SHARK-dive score does not strictly represent the similarity between the two sequences, although a higher SHARK-dive score generally reflects a higher confidence of homology. Interesting candidates can then be further analyzed using the accompanying notebook to visualize and locate the similar sequence features/regions between the two sequences. SHARK-dive is currently usable as a Python3 package with an accompanying user manual.

It is important to note that SHARK-dive was developed as a homology classifier akin to BLAST/HMMER. It does not explicitly aim to classify IDR functions, but rather to assess the homology between IDRs and other difficult-to-align sequences. Admittedly, SHARK-dive cannot determine whether the predicted homology relationships are by shared function (i.e., analogs) or by a shared evolutionary ancestry (i.e., orthology, or evolutionary homology). For example, while SHARK-dive can detect homology between the *S. cerevisiae* Ste50 IDR and orthologous yeast IDRs (*Candida glabrata* and *Lachancea kluyveri*) as well as to analogs (*S. cerevisiae* Stp4 and Pex5), it is unable to discern between the two. Instead, this will require further functional and phylogenetic analysis (e.g., by synteny).

The approach of SHARK-dive as a homology predictor is orthogonal to direct IDR function prediction by tools such as DEPICTER2 (84) and DisoFLAG (85), as well as more specialized predictors such as CoMemMoRFPred (86) for membrane-binding molecular recognition features and HybridDBRpred for DNA-binding regions (87), which can assess whether particular IDRs, or even specific residues, are associated with a functional category. As such, SHARK-dive is agnostic to the particular function, instead functioning purely as a homology predictor. Whereas a drawback of homology-based functional inference is the requirement that the function of at least one IDR is known, possible synergy nonetheless exists between a function classifier and a homology classifier such as SHARK-dive. Where the former can provide de novo functional prediction given a sequence, the latter can then be used to identify possible functional homologs, especially if any experimental evidence highlights a more specific function. The homology prediction approach of SHARK-dive and its ability to highlight regions of sequence similarity may be useful for subsequent mutagenesis studies to uncover the sequence determinants required for that function, which can then be further used to train and improve the performance of the IDR function predictors.

SHARK-dive is also inspired by recent efforts that seek to identify clusters of IDR analogs with similar functions but differs significantly in its approach. These analyses (14, 88, 89) rely on feature-based comparisons which assess IDR similarity and classify/cluster IDRs based on their sequence “molecular features” where each IDR is represented as a feature vector reflecting the conservation of specific characteristics across a set of extant orthologs, where conservation is detected as deviations from computationally simulated orthologs via an in silico evolution protocol (14, 90). These features can include the presence of particular SLiMs/motifs or bulk properties such as net charge and hydropathy, which can be determined without relying on alignments. Accordingly, Zarin *et al.* demonstrated that this approach (14, 88), which compares the feature vectors (referred to as “evolutionary signatures”) via cluster analysis, can identify clusters of IDRs where function could be complemented within the cluster, as illustrated by the *S. cerevisiae* Ste50 and Cox15 examples, and this has recently been applied even to the human proteome (91) and adapted to the *Drosophila* proteome (38). The feature vector approach is also adopted by FAIDR (Feature Analysis of IDRs), which aims to predict protein function given a predefined IDR feature vector. These investigations mark significant progress in the direction of alignment-free studies of IDR homology. However, a potential challenge for this approach is its generalizability and scalability, particularly as our understanding of IDR biology grows and more molecular features are found to be under functional conservation. Such features may also be species dependent, therefore Zarin et al. have cautioned against directly comparing individual evolutionary signatures to assess homology (14). Furthermore, Singleton and Eisen reported the relationship between conserved features and specific biological functions is more nuanced in multicellular organisms than in yeast, likely due to more complex regulation in multicellular organisms (38). Accordingly, we see SHARK-dive as a bottoms-up approach to homology assessment that should scale well since it does not require extensive a priori feature selection.

In fact, not only does SHARK-dive extend upon such previous efforts to facilitate studies of IDR biology, it may also reciprocally enhance the performance of existing IDR function prediction tools. For example, the performance of FAIDR is impacted by the retrieval of extant IDR homologs (88, 91), since it requires a list of distantly related homologous sequences as a starting point. This means that certain IDRs may currently not be amenable to FAIDR analysis due to insufficient information on evolutionarily related distant homologs. As such, SHARK-dive-retrieved homologs can be included as inputs to provide sufficient evolutionary depth and expand the coverage of IDRs available for FAIDR analysis, thereby complementing IDR function prediction tools.

Ultimately, SHARK-dive is built upon the underlying principles and successes of these previous studies: integrating their ideas of using alignment-free analysis for IDRs with an extended alignment-independent definition of orthologous IDRs for homology prediction. We hope that the unalignable orthologs dataset may be further adopted by IDR annotation tools and that SHARK-dive can contribute to the sustained development of a systematic repository of IDR functions, thereby laying the groundwork for facilitating the study of sequence–function relationships in disordered, difficult-to-align regions.

## Materials and Methods

### Disorder and Unalignability in the Protein Universe.

Disorder and unalignability in the protein universe (SwissProt 2022-03 release) was quantified as the fraction of disordered residues as per IUPRED2a (disorder score ≥0.4), and and domain residues according to Pfam annotations respectively. The mean per-family degree of disorder in Pfam seed sequences was also calculated as the mean IUPRED2a sequence disorder score of the sequence across seed sequences of each family. Concurrently, the prevalence of sequences in SwissProt that contains 1) a disordered region and 2) a Pfam domain was also assessed.

### Development of the SHARK Algorithm and SHARK-scores.

SHARK is a *k-*mer-based method to assess the similarity between two sequences, which incorporates amino acid physicochemical properties (in the form of Grantham similarity matrix (47)) to assess the similarity between the *k*-mers. Quantifying the similarity between all the *k*-mers of both sequences to yield a *k-*mer similarity matrix and then aggregating either the best match or all sufficiently similar matches above a similarity threshold *x* yields the SHARK-score (best) and SHARK-score (T = *x*) variant, respectively. SHARK-scores were then benchmarked against local alignment (using the Smith-Waterman algorithm, SW) and other existing alignment-free metrics [Euclidean Distance, Normalized Google Distance (92), and the cosine-similarity-based Similarity Ratio (93)].

### Training and Performance Evaluation of SHARK-dive.

SHARK-dive is a homology classifier which requires 10 input features, *k*-mer scores from *k*=1 to *k*=10. SHARK-dive is a 10-fold ensemble gradient-boosted decision tree classifier trained on a set of orthologous sequence segments (orthology was defined by the OMA browser (49), heretofore known as a sequence family) where a member sequence is functionally annotated in DisProt (48, 94), where the evolutionary homology between sequence segments is defined purely by their surrounding domains and the overall domain architecture of the protein without requiring multiple sequence alignments. The performance of SHARK-dive was benchmarked on a withheld testing set of sequence families against 1) local alignment with commonly used matrices such as BLOSUM62 and PAM30 as well as a series of disorder-specific matrices [EDSSMAT, (56)], and 2) widely used alignment-based homology prediction tools BLAST (8) and HMMER (80).

### SHARK-dive Concordance to Literature-reported IDR Homologs and Hypothesis Generation.

The ability of SHARK-dive, BLAST, and HMMER to predict evolutionary homology and functional analogy was reported on a set of IDR swap experiments (14, 57), where an endogenous IDR was replaced with highly diverged orthologous IDRs or with dissimilar IDRs with low sequence identity. SHARK-dive was then used to search for candidate IDR homologs to the FUS PLD (residues 1-211) and FUS RBD (residues 212-536) in the proteome in a blind query-database search.

Please see *SI Appendix*, *Materials and Methods* for a full description.

## Supplementary Material

Appendix 01 (PDF)

## Data Availability

Code and sequences data have been deposited in the gitlab repository and SHARK-dive supporting datasets. The codebase and readme files can be found at https://doi.org/10.5281/zenodo.13847324 (95). Further, we provide jupyter notebooks to aid easy interpretation and visualization of SHARK-dive scores: https://git.mpi-cbg.de/tothpetroczylab/shark/-/tree/master/notebooks (96). All supplementary datasets are available at https://doi.org/10.17617/3.DIAVNC) (97). SHARK is available as a pip installable Python package at https://pypi.org/project/bio-shark/1.2.1/.
