# Supplementary material for: SHARK enables sensitive detection of evolutionary homologs and functional analogs in unalignable and disordered sequences
Source: Proc Natl Acad Sci U S A. 2024 Oct 9;121(42):e2401622121. doi: 10.1073/pnas.2401622121 (PMC11494347; doi:10.1073/pnas.2401622121)
Supplement: Supplementary file 1 — Appendix 01 (PDF) [file pnas.2401622121.sapp.pdf]

Supplementary Information

SHARK enables sensitive detection of evolutionary homologs and functional analogs in unalignable and disordered sequences

Chi Fung Willis Chow[1,2,3], Soumyadeep Ghosh[1,2], Anna Hadarovich[1,2], Agnes Toth-Petroczy[1,2,3]*

Affiliations

1. Max Planck Institute of Molecular Cell Biology and Genetics, Pfotenhauerstrasse 108, 01307 Dresden, Germany
2. Center for Systems Biology Dresden, Pfotenhauerstrasse 108, 01307 Dresden, Germany
3. Cluster of Excellence Physics of Life, TU Dresden, 01062 Dresden, Germany

*corresponding author, toth-petroczy@mpi-cbg.de

Supplementary Online Methods

Supplementary Figures 1-28

Supplementary Tables 1-9

Supplementary Datasets 1-6

Supplementary References

Data and code availability

The code base and readme files can be found at https://git.mpi-cbg.de/tothpetroczylab/shark. Further, we provide jupyter notebooks to aid easy interpretation and visualization of SHARK-dive scores: https://git.mpi-cbg.de/tothpetroczylab/shark/-/tree/1-version-release-workflow/notebooks . All supplementary datasets are available at https://doi.org/10.17617/3.DIAVNC

Supplementary Online Methods

UniProt-wide analysis of disorder and definition of alignability

All 568002 Swiss-Prot sequences in the 2022-03 release of UniProt were retrieved (1). For each sequence, we mapped the domains (defined by the alignment coordinates to the profile HMM) using HMMscan using the Pfam35.0 database with the gathering thresholds as a cutoff (2). Sequences not longer than 10 residues were removed. Each residue was then annotated as ordered/disordered or domain/non-domain depending on its IUPred2A disorder score (long option) (3, 4), and its Pfam annotation respectively. We use a threshold of 0.4 to account for disordered residues that have a propensity for disorder-to-order transitions, and define unalignable residues as those that do not map to Pfam domains (non-domain).

Extraction of IDRs

Reference proteomes were obtained from the 2022-05 release of UniProt for 7 organisms: *H. sapiens, M. musculus, E. coli, S. cerevisiae, A. thaliana, D. rerio, D. melanogaster* (Table S8). Each sequence was analyzed using the disorder predictor IUPred2a (long) (3), with smoothing applied to residue-specific disorder values using a Savitzky-Golay filter (moving average window size=9, polynomial degree=3). Setting the disorder threshold at 0.4, consecutive disordered residues were concatenated into IDR segments. To identify the number of proteins with significant disorder in Swiss-Prot (Figs. S1 with IUPred, S4a with flDPnn), the length of the longest IDR segment present was chosen. For IDR homology searches in proteomes, only ≥10 amino acid IDR segments without unresolved or non-canonical amino acids were considered.

Development of the alignment-free SHARK algorithm and SHARK-scores

In summary, SHARK-scores compares sequences using 6 main steps. The first 4 steps form the core of the SHARK algorithm:

1) Each sequence (query *Q* and target *T*) is decomposed into a *k*-mer vector, where *k* represents the length of the peptide. Each *k*-mer vector encodes the identity of the *k*-mer and the frequency (q_i or t_j) at which it occurs in the sequence, where *i* and *j* represent each unique *k*-mer in sequence *q* and *t* respectively.
2) The similarity between 2 amino acids (D') is derived from an amino acid physicochemical distance matrix (*G*) (in our case the Grantham's Distance matrix(5)). To convert from a distance (i.e. a measure of dissimilarity) to a similarity measure, the distance values D' are inverted and min-max normalized to give *k*-mer similarity score D:
$$D = 1 - \frac{D' - \min(G)}{\max(G) - \min(G)}$$
3) Between two *k*-mers *i* and *j* of identical length, the similarity *w* is calculated as
$$w_{i,j} = \frac{\sum_p D_p}{k}$$
i.e. the mean physicochemical similarity across each amino acid position (p) of the *k*-mers. As such, *w*=1 for identical amino acids and *w*=0 for the most dissimilar pair (Cysteine-Tryptophan).
4) Between two sequences, all k-mers are compared against each other to form a similarity matrix (*M*), which gives the *k*-mer similarity (*w*) between the unique *k*-mer pairs of both sequences.
5) For each *k*-mer pair (*i,j*) in *M*:
   a) All but the most similar *k*-mer in the other sequence are filtered out (w set to -1) to give a sparse matrix. Each non-zero element in the matrix is multiplied by the frequency product in both

sequences $q_i \cdot t_j\ (where\ w > -1)$ and weighted (divided) by a length correction (LD) function, defined as:

$$LD(q_i, t_j) = \left(\frac{\sqrt{2({q_i}^2 + {t_j}^2)}}{q_i + t_j} \right)$$

The LD function serves to account for the difference in frequencies between k-mers. In the case where multiple k-mers have equivalent similarity, the most similar k-mer is chosen based on the lowest LD value. Finally, this is summed across the entire matrix and normalized by the sum of the frequency products of non-filtered k-mers in both sequences , $\sum q_i \cdot t_j\ (where\ w > -1)$, to give the best-match SHARK-score (SHARK-score (best)). Identical sequences will score 1.

b) All k-mers not exceeding a similarity threshold (x) are filtered out (w set to -1) to give a filtered matrix. Resultingly, each k-mer (i or j) contains only sufficiently similar matches to itself, represented by values > -1 in its row/column (row for query k-mers i, column for target k-mers j). For each k-mer, its row/column score is then aggregated by calculating the weighted average similarity across the row/column, again using LD to factor in the difference in k-mer frequencies between the k-mer and the total number of matches sufficiently similar k-mer matches in the other sequence. In other words, each k-mer is compared to a "composite" k-mer that represents the overall similarity of the matches:

$$row\ score = \frac{q_i * \frac{\sum_j (w_{i,j} * t_j)}{\sum t_j}}{LD(q_i, \sum t_j)} \qquad (for\ all\ j\ where\ w_{i,j} > x)$$

$$column\ score = \frac{t_j * \frac{\sum_i (w_{j,i} * q_i)}{\sum q_i}}{LD(t_j, \sum q_i)} \qquad (for\ all\ i\ where\ w_{j,i} > x)$$

All row and column scores are summed (i.e. for all unique k-mers across both sequences) and then normalized by the total number of k-mers across both sequences, $\sum q_i + \sum t_j$, to give the filtered SHARK-score (**SHARK-score (T=*x*)**. We note that the filtered score does not guarantee that identical sequences get the maximum score of 1, but is designed to fully capture the correspondences between all sufficiently similar k-mers between 2 sequences.

6) The matrix is invariant to which sequence is used as query and target (the similarity matrix M is simply transposed), ensuring that scores are symmetrical.

Curation of disordered Pfam families (alignable-disorder dataset)

Pfam is a repository of homologous domain sequences grouped into families(2). Sequence homology is assessed by concordance to an alignment-based Hidden Markov Model built from manually-curated seed sequences.

To select for the most disordered Pfam families in Pfam 34.0 (released March 2021)(2), the disorder in the 90% non-redundant seed sequences of each family was analyzed using IUPred2a (long option)(3, 6). For each sequence, the mean disorder score across all positions (mean sequence disorder) was calculated; redundant sequences (>90% identity) and sequences where the seed and the full alignment differed were discarded from analysis. For each family of seed sequences, the mean sequence disorder across all seed sequences were averaged to give the mean seed disorder.

1440 Pfam families with mean seed disorder >0.5 were selected for further analysis, and their domain sequences (full alignment) were obtained from Pfam. CD-HIT (7) was applied to filter out sequences with more than 50% identity; families with fewer than two sequences were also ignored since sample standard deviation cannot be calculated, 1374 disordered families remained. From these families, the mean sequence disorder was calculated for all domain sequences of that family and averaged to give the mean family disorder; the variation in mean sequence disorder was also calculated as the standard deviation of family disorder. Within these 1374 families, a dataset was curated containing families in the highest 25% of mean family disorder and lowest 25% standard deviation of family disorder, ensuring that most (if not all) sequences are disordered. Finally, sequences shorter than 10 amino acids or containing unresolved amino acids ("X") were removed, giving 2583 sequences across 143 Pfam families (Dataset S1).

Curation of unalignable, orthologous sequences from DisProt (unalignable-ortholog dataset)

Entries with functional annotations were curated from the DisProt database (2022-03 release)(8), this includes all entries with annotated molecular function/disorder functions in the 'term_namspace' column. Orthologous proteins across the tree of life were identified using the OMA orthology database (Dec 2021 release)(9). Orthologs in multiple orthology groups, as well as sequences with ambiguous OMA identifier – UniProtID or sequence – OMA identifier mappings, were excluded.

Using HMMscan against the Pfam35.0 database with the gathering thresholds as a cutoff, we mapped the alignable domains (defined by the alignment coordinates to the profile HMM) onto each sequence to obtain its domain architecture. Domains were mapped sequentially to avoid overlap, with the most significant domains (in ascending order of sequence E-value) mapped first. Subsequent domains that overlap with mapped domains were excluded, ensuring that each residue is only mapped to one domain. Each protein is thus represented by its domain architecture consisting of an N-terminus, followed by all mapped Pfam domains linked by non-domain sequences, and ending with a C-terminus (Fig. 3a). Using the protein entry curated in DisProt as a reference, we selected only for orthologs with an identical domain architecture. This allowed correspondence to be established between each non-domain segment across orthologs, solely based on their surrounding domains. We then mapped the amino acid coordinates (start and end positions) of each functional IDR region onto the sequence. Where the IDR overlaps with a non-domain segment, we extracted the corresponding non-domain segments in orthologs flanked by the same set of domains to give a family of orthologous unaligned segments (henceforth referred to as a sequence family). In rare cases (0.26% of sequences in the final dataset across 2.2% of sequence families) where the DisProt-annotated IDR regions of the reference entry are fully mapped by Pfam domains, non-Pfam annotated regions in orthologous sequences were still extracted since they still correspond to the same sequence family (flanked by the same set of domains). In cases where the DisProt function annotation spans multiple non-domain regions, we extracted each sequence family separately.

All sequence families were aggregated, segments not longer than 10 amino acids or containing unresolved amino acids were removed, and filtered for <50% identity with CD-HIT (7). Due to the requirement during our dataset curation that Pfam domain regions must be non-overlapping and sequentially mapped by sequence E-value, we performed a final HMMscan on sequences and removed 183 sequences (1.4%) that contained Pfam domains, ensuring that our sequences are fully non-Pfam aligned. This yielded a set of 12602 sequences across 717 families of orthologous unaligned segments (Dataset S2). To ascertain the disorder content of these segments, these sequence segments were analyzed with IUPred2a (long) and flDPnn (3, 10).

Ded1p orthologous N- and C- termini sequences (Ded1p dataset)

Eukaryotic orthologs belonging to the eukaryotic orthologous group of *S. cerevisiae* Ded1p (UniProt ID P06634) were extracted using the EGGNOG orthology database (v5.0) and manually curated. From the multiple sequence alignment of the orthologs with MAFFT (v7.453)(11), the N- and C- termini IDRs were obtained according the helicase core domain boundaries of the *S. cerevisiae* sequence, defined as positions 99-535 according to alignment to the human ortholog DDX3X for which a crystal structure is available (PDB 2I4I, 4PXA, 5E7I). The MSA was manually curated and removal of redundant, highly similar (>95% identity) sequences was performed using CD-HIT. A final quality control step saw the removal of <10 amino acid N- and C- terminal sequences. Concordantly, an erroneous yeast paralog was manually removed and replaced with the human DED1 ortholog DDX3X. This gave a set of 268 orthologs each with an N- and C- terminal IDR (536 sequences in total). Sequences are visualized in Figure 1a using AliView(12), and column-wise amino acid physicochemistry conservation calculated using Jalview's built in tool(13, 14).

Performance of homology assessment

All-vs-all sequence comparisons were performed and the ability of the various algorithms to classify homologs was assessed by plotting the receiver operating characteristic (ROC) and precision-recall (PRC) curves.

In the case where the number of true positive (homologous, TP) and true negative (unrelated, TN) sequence pairs are similar such as the Ded1p dataset, the auROC provides a more balanced measure. For the alignable-disorder and unalignable-ortholog test datasets the overall performance is reported as the integrated area under the precision-recall curve (auPRC). This is due to the class imbalance from the all-versus-all comparison, which yields a far greater number of unrelated sequence pairs than true homologs. The auPRC quantifies the ability to detect true positives whilst minimizing false positives in the prediction and is more suitable in situations with a class-imbalance.

Besides SHARK scores and the SHARK-dive model, the following alignment-free metrics were also compared: Normalised Google Distance (NGD) (15), Similarity Ratio (Simratio) (16) and Euclidean distance. NGD and Simratio was reported as the best performing metrics in an alignment-free homology assessment benchmark (17), whilst Euclidean Distance is a commonly used distance metric. The alignment-based algorithms HMMER, BLAST and Smith-Waterman local alignments (with Biopython pairwise2 module) were also benchmarked.

Alignment-free scores can reach maximum dissimilarity and lose sensitivity to remote homologs with longer *k*-mers because there are no similar/identical *k*-mers shared between homologs. Accordingly, we define the 'area of non-sensitivity' as the region in which the scores are unable to distinguish between true homologs and unrelated sequences, calculated via the trapezium rule between the final recall point (recall=1.0) and the previous recall point. For the alignable-disorder dataset, a threshold of 0.003 was set such that only *k*-mer lengths and scores with area of sensitivity< 0.003 are considered suitable and subsequently used, so as not to over-estimate their precision-recall performance (Fig. S7).

To assess threshold-specific performance: for local alignment scores with Smith-Waterman, the threshold of homology was chosen based on the optimal F1 score on the withheld test set, specific to each substitution matrix and gap penalty parameters. Threshold for existing alignment-based homology search tools (BLASTp, pHMMER) was chosen based on default sequence E-values and bit-scores used. Performance of stricter (i.e. lower) E-values are also reported (Table S4). Following selection of the threshold, threshold-specific performance metrics such as sensitivity/recall, specificity, accuracy and F1 were also calculated (Table S5). PID-stratified sensitivity and F1 performance is also reported.

Protein-protein BLAST (BLASTp) was performed using both standard (blastp) and short (blastp-short) options. Sequences databases were generated from FASTA files using the makeblastdb command, which was then used to search against by query sequences (in FASTA files). To maximize sensitivity and detection of remote homologs the E-value threshold was set to 10^4. Default parameters were used for BLAST searches, and we also included the following substitution matrices and gap penalties (Table S). Note, that we use BLAST's definition of gap cost. Specifically, given a gap of length *n*:

$$Gap\ cost\ (n) = G + Ln$$

where G is the gap opening cost and L is the gap extension cost.

For HMMER the reporting and inclusion E-value thresholds (-E and -incE) were set to 10^{14} once again to maximize sensitivity, alongside the --max option that turns off sequence filters and runs the full postprocessing on all targets to ensure maximum sensitivity.

Unless otherwise specified, local alignment was performed using Smith-Waterman dynamic programming with the indicated substitution matrix with parameters in accordance with default values and definition of gap costs used in BLASTp searches (Table S1). The PFASUM70 matrix was rounded to the nearest integer per Keul *et al.* (18). Other matrices were use as-is.

SHARK-dive model feature selection and training

Feature selection of the SHARK-dive model was based on the performance of individual scores on the alignable disorder dataset. We selected 10 features, one scoring method per *k*-mer length from 1-10 (Table S2).

A 70:20:10 train-validation-test split of the unalignable ortholog dataset by family was performed. We split sequences at the family level to ensure that test dataset sequences are not homologous to sequences used in training. We further split the validation set in half for early stopping to prevent overfitting (validation 1) and hyperparameter tuning (validation 2), respectively. We verified that IDRs were retained in all splits during the train-test split by both IUPred (Fig. S9) and flDPnn (Fig. S10). The final numbers of sequences and sequence families in each split are detailed in Fig. S9e.

To account for the variability in family size and sequence lengths, we developed an ensemble model with 10 sub-models. Each sub-model is a gradient-boosted decision tree classifier trained on a particular subset of families. Accordingly, the training dataset was split into 10 overlapping folds by orthologous segment family. Each fold was held out once for cross-validation (cross-validation fold), and the 9 remaining folds combined into a training fold used to train the model. Accordingly, this gives 10 sub-models, each trained on a large training fold from the combination of the 9 folds with the stated hyperparameters. The validation1 sequences were introduced as an early-stopping dataset to prevent overfitting, with training stopped when the performance on the validation1 sequences does not increase in the following 100 rounds. All training and cross-validation folds, as well as the early-stopping validation1 dataset, were balanced for number of homologs and number of unrelated sequences (same number in of TP and TN). We verified that the choice of TN does not affect model performance (Fig. S26a,b). To tune the CatBoost(19) model hyperparameters, we tested 250 combinations of three hyperparameters via a grid search (Dataset S4) and then selected the best performing model based on F1-score on the validation2 dataset. This corresponded to a number of weak learners of 700, maximum depth of 8 and learning rate of 0.1. The cross-validation performance of each sub-model (i.e. each training fold) on its corresponding validation fold is shown in Figure S27. We further note that the feature importance of each sub-model has similar trends (Fig. S26c). The final SHARK-dive score is the unweighted mean prediction across all 10 sub-models.

Percentage Sequence Identity (PID) calculation

Sequences were globally aligned using Biopython pairwise2 module, with BLOSUM62 matrix and standard gap penalties (Table S1). End gaps were not penalized. Percentage sequence identity was defined as fraction of identical residues in the alignment (aligned residues + gaps within the alignment i.e. the length of the alignment), i.e.

$$PID = \frac{aligned\ residues}{aligned\ residues + internal\ gaps}$$

and lies in accordance with the Doolittle definition(20, 21).

IDR replacement/functional rescue validation

Sequences were obtained from the publications reporting such IDR replacement experiments, directly from supplementary information or from Yeast Genome Browser(22)-UniProtID mappings(23, 24). For all tools SHARK-dive, pHMMER and BLAST (BLOSUM62), a database comprising all replacement IDRs as well as the WT IDR was curated, and a WT-versus-all comparison performed. To account for the smaller size of the sequence database against which sequences are searched (<10 sequences versus the ~1800 sequences for the unalignable-ortholog test dataset), E-values for homology detection are accordingly adjusted to 0.05 since they depend on the size of the database (we adopt BLAST's definition of database size as the number of residues in the database(25)). DEDAL homology detection/classifier tool was used, which outputs homology detection logits (log-odds score): the threshold of 0 is used, corresponding to a probability of homology =0.5. Multiple sequence alignments were performed using MUSCLE (v3.8.31) unless otherwise indicated(26).

Runtime and memory analysis

Runtimes and memory requirements were calculated on a server equipped with Intel(R) Xeon(R) Silver 4208 CPU @ 2.10GHz (32 logical cores) with 98GB RAM, with 10 simultaneous threads (i.e. on 10 logical cores) allowed for a database search of one sequence (195 amino acids long) against 100-1000000 SwissProt sequences (minimum length of 10 amino acids). Since each *k*-mer score can be calculated independently, SHARK-dive can be parallelized to calculate 1 *k*-mer score/core, and using a final aggregation step to calculate the homology predictions. Results of the runtime/memory analysis are shown in Figure S28.

flDPnn disorder prediction

flDPnn(10) was used to assess disorder in parallel to IUPred. The default residue-wise binary disorder prediction of flDPnn was used to determine if a residue was considered disordered and to calculate the fraction of disordered residues in a sequence. The flDPnn score was used to assess the mean disorder (mean flDPnn score) of a sequence.

Since flDPnn is much slower to run than IUPred, the SwissProt-wide analysis of disorder and unalignability was run on a 90% non-redundant (CD-HIT maximum identity 90%) set of sequences of between 10-5000 amino acids in length (due to flDPnn's maximum length limit), using the default residue-wise binary disorder prediction. The rest of the analysis is identical to that of IUPred (see Methods, UniProt-wide analysis of disorder and definition of alignability).

Sequence helix content

Helical behavior in the unalignable orthologs dataset was determined using the Agadir (27) webserver (http://agadir.crg.es/), which reports the helix content of each sequence (Agadir Score). Default parameters, corresponding to pH 7, 278K, 0.1 ionic strength and no N-/C- terminus protection were used.

Supplementary Figures

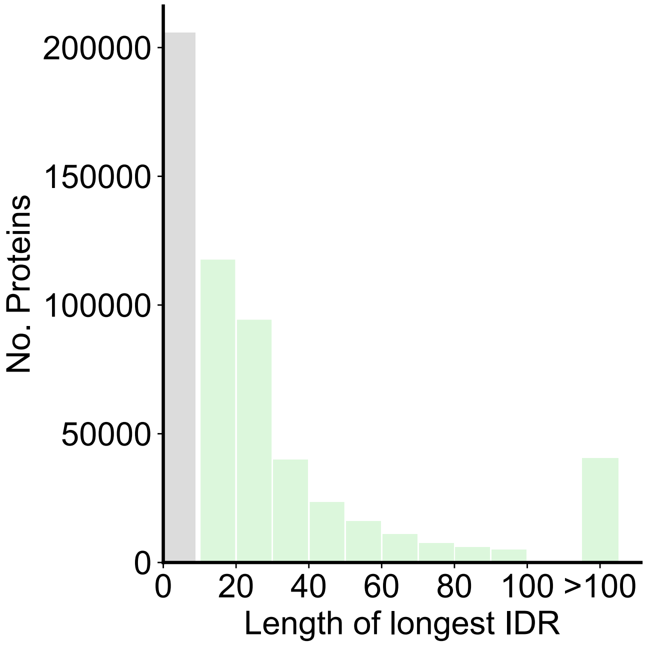

Figure S1. Most proteins contain a disordered region. Distribution of the longest IDR found in each curated Swiss-Prot protein (*n*=567,192). 63.7% of proteins contain an IDR of at least 10 amino acids in length (green).

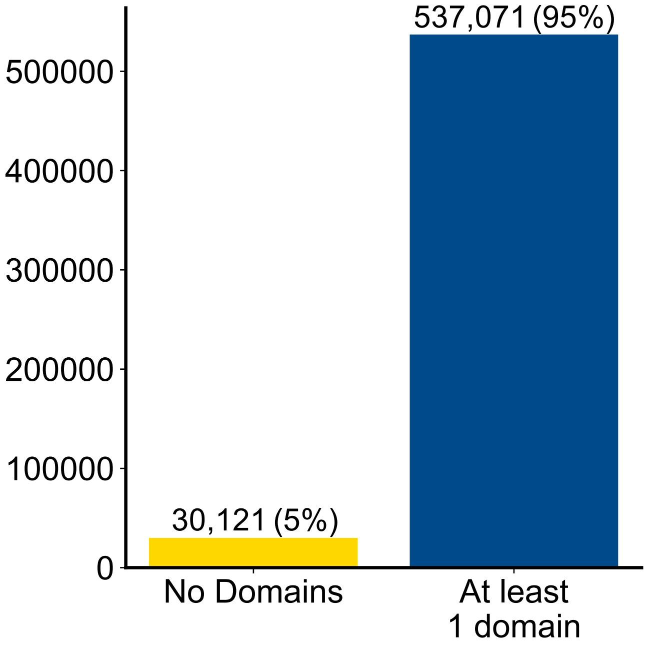

Figure S2. ~5% of SwissProt sequences lack any Pfam domain annotation. The number of protein sequences with no Pfam domain annotation (yellow) and at least one Pfam domain annotation (blue) are shown. Sequences based on the UniProt 2022-03 release, with <10 amino acid long sequences removed (see Methods: UniProt-wide analysis of disorder and definition of alignability). Pfam domains mapped with Pfam35.0.

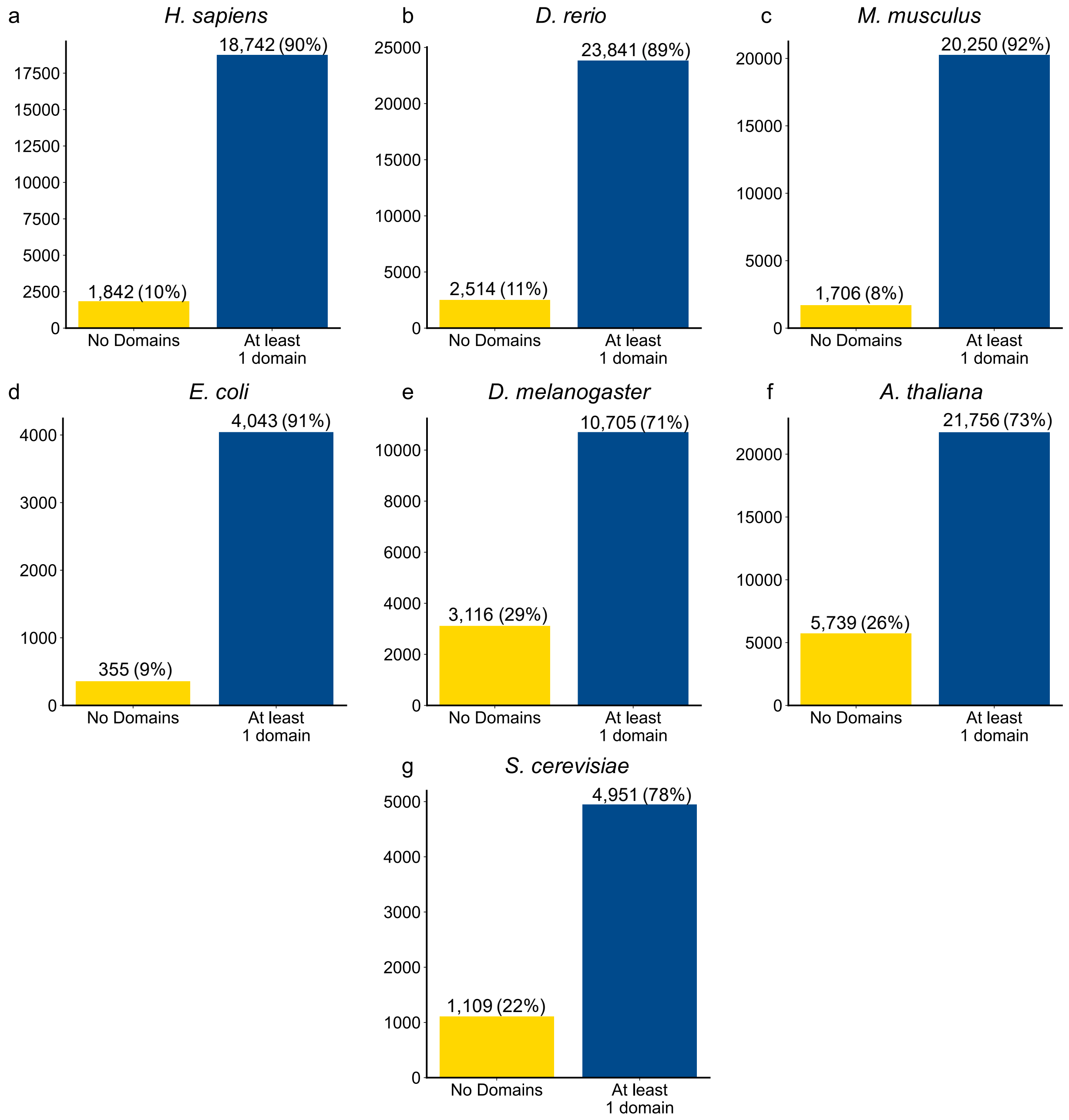

Figure S3. Reference proteomes of 7 model organisms contain varying degrees of sequence Pfam coverage. The number of protein sequences with no Pfam domain annotation (yellow) and at least one Pfam domain annotation (blue) shown for different organisms. Sequences based on the UniProt 2022-05 reference proteomes release, with <10 amino acid long sequences removed (see Methods: UniProt-wide analysis of disorder and definition of alignability). Pfam domains mapped with Pfam35.0.

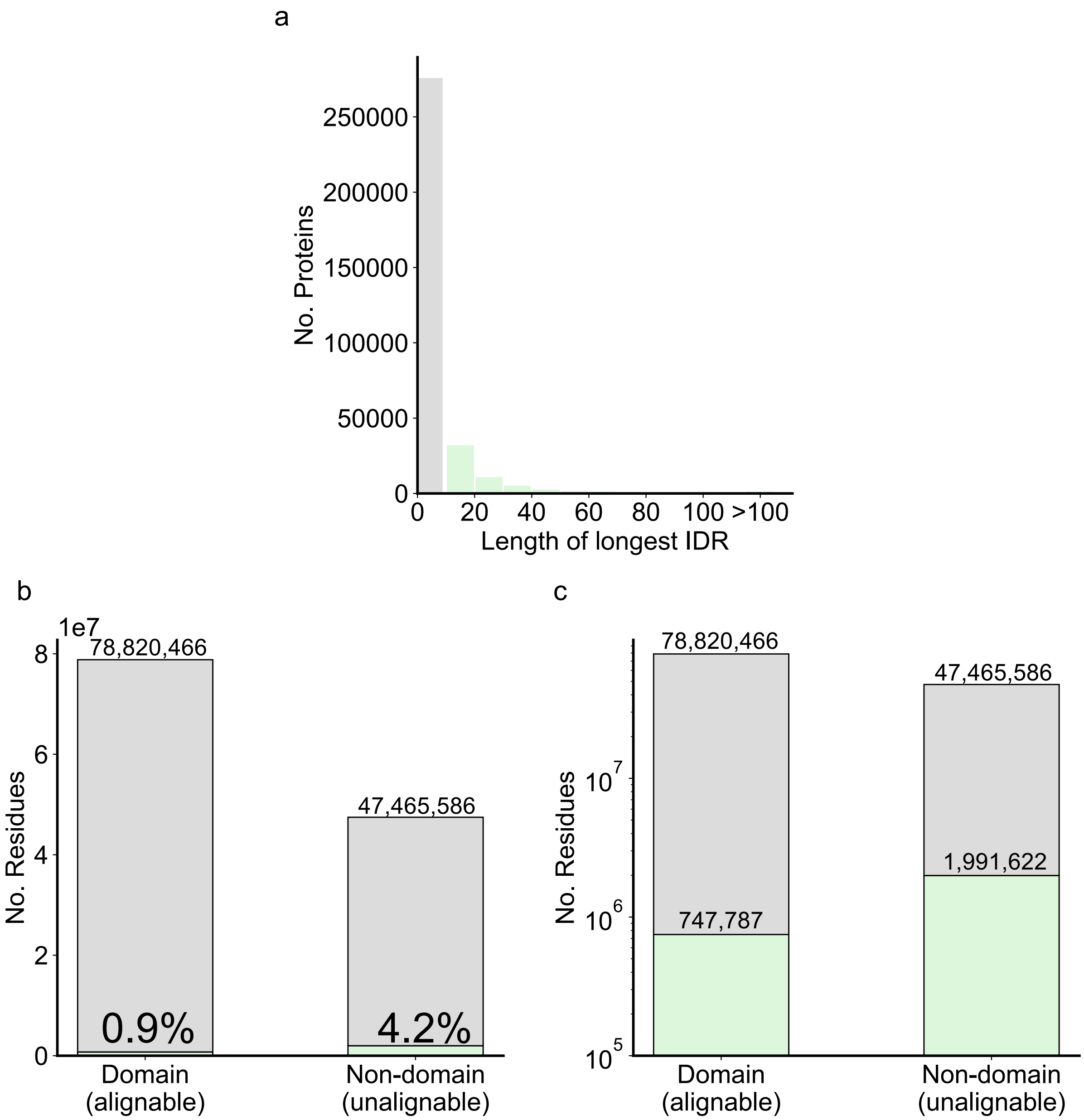

Figure S4. flDPnn predicts fewer long disordered regions across SwissProt, but disordered residues are ~4x more prevalent in non-domain regions than in Pfam domains. a) flDPnn analysis of disorder in SwissProt (minimum 10 amino acids, 90% non-redundant) predicted fewer proteins with ≥ 10aa IDRs (16.7%). However, of the sequences evaluated, non-domain regions contain a larger fraction of disordered residues than ordered regions (b,c), with a 4x greater fraction of disordered residues in non-domain regions than in Pfam domains. This is congruent with the results from the analysis performed using IUPred (Fig. 1b). N.B. b,c are identical except for a log-scale on the y-axis.

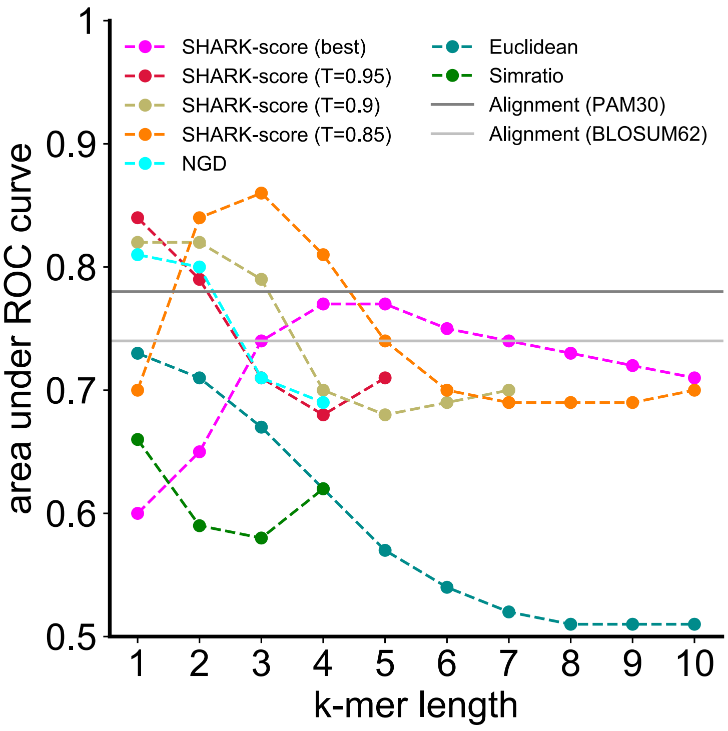

Figure S5. For a set of Ded1p orthologs, SHARK-scores outperform alignment and existing alignment-free metrics in differentiating sequences which have opposing functional effects. In order to assess the ability of SHARK-scores in identifying functionally similar sequences, we performed a proof-of-concept test on a set of eukaryotic Ded1p orthologs. Work by Iserman *et al.* identified opposing functions of the N- and C- terminal IDRs on phase separation behavior in Ded1 (Fig. 1a)(28). Specifically, the N-terminal IDR inhibits heat-induced phase separation of Ded1 whilst the C-terminal promotes condensate formation via protein-protein interactions. Since these functions are likely under selection and thus conserved between orthologs, a set of Ded1p N- and C- terminal IDR orthologs were curated.

We assessed the ability of different algorithms to capture the homology between IDRs from the same terminus and observed that alignment-free metrics generally outperform Smith-Waterman dynamic-programming algorithm (henceforth referred to as local alignment). In addition to the commonly used Euclidean distance, we also tested the performance of Normalized Google Distance (NGD) and Similarity Ratio (Simratio), which had previously been reported to be the best performing algorithms in an alignment-free remote homology detection benchmark(17). Between k=1 (amino acid composition) and k=10, SHARK-score (T) consistently offered the best overall performance according to the area under the receiver operating characteristic. By tuning the similarity threshold T, SHARK-scores were able to achieve high performance at higher k's where existing metrics begin to deteriorate. A small benchmark notwithstanding, this indicated the promise of SHARK-scores in assessing homology between IDRs. For each algorithm, only k-mer lengths where the auPRC would not be over-estimated due to the lack of similar/identical k-mers between homologs were plotted (see Methods).

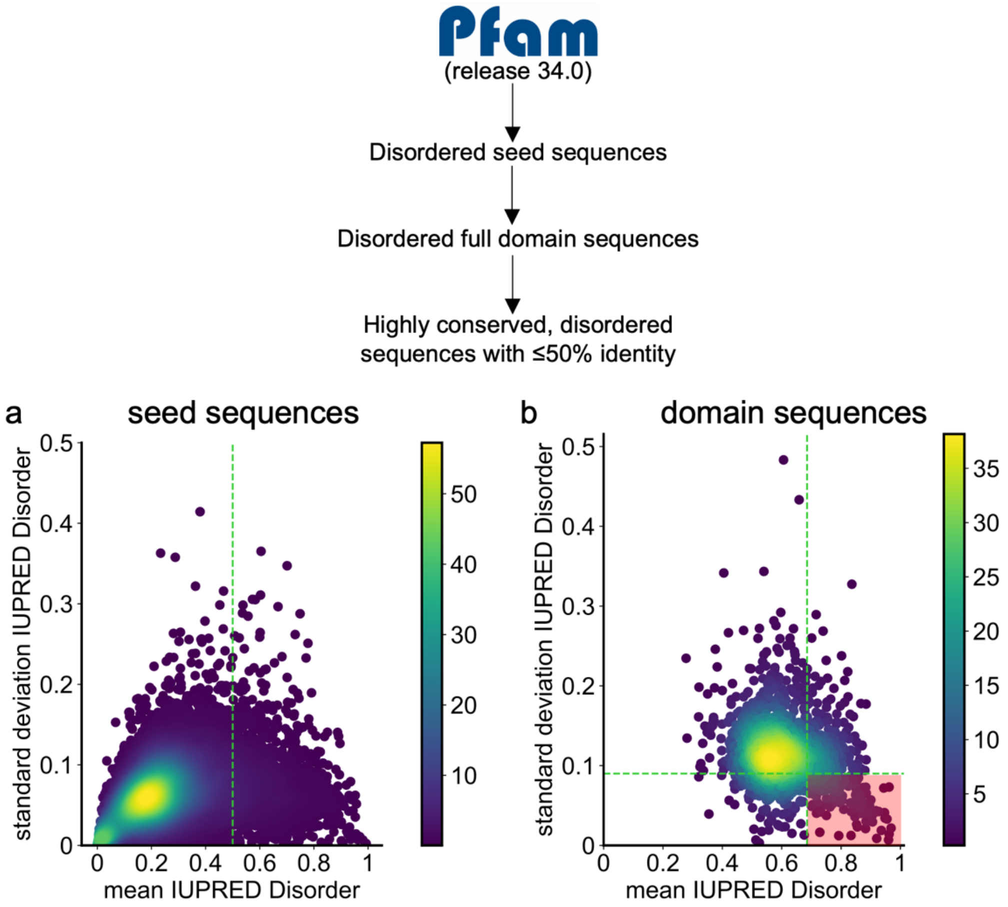

Figure S6. Overview of the curation of the most disordered Pfam families (alignable-disorder dataset). a. Density plot of the mean and standard deviation of seed family disorder of Pfam family seed sequences in the 18188 families analyzed. b. Density plot of the mean and standard deviation of full family disorder of Pfam family domain sequences at max 50% identity. Shaded in red is an area of highly conserved disorder, and contains only families that are in the 75th percentile of mean family disorder and within the 25th percentile of standard deviation, to ensure that all sequences of the family are consistently disordered. This corresponds to 143 families that were included in the alignable-disorder dataset.

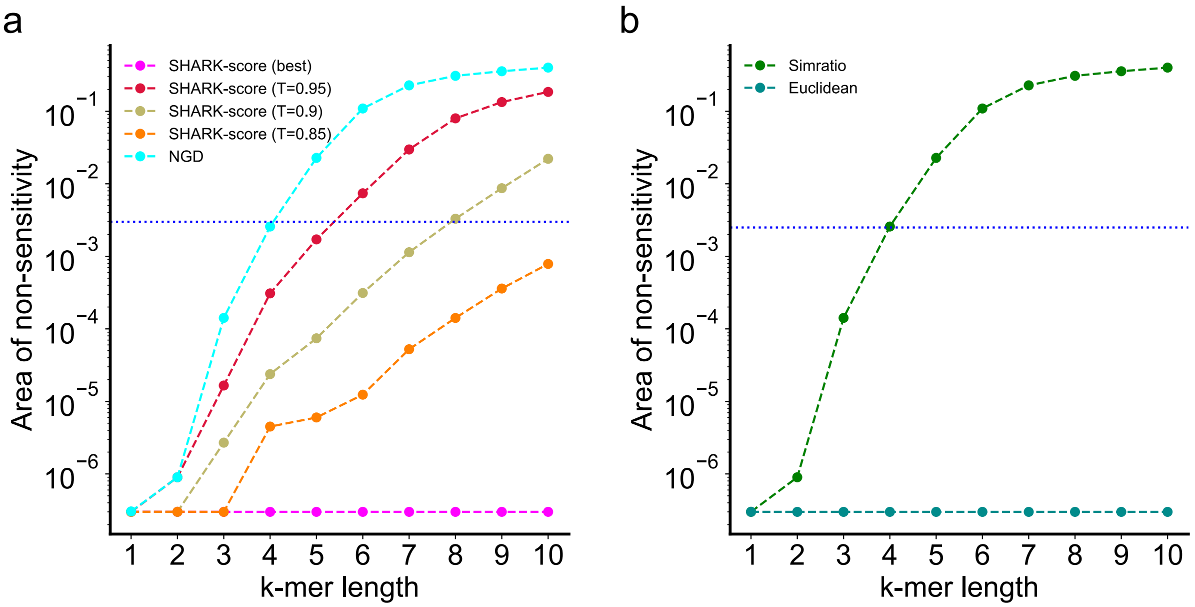

c

Alignment-free algorithm	Maximum *k*-mer length
SHARK-score (best)	10
Normalized Google Distance (NGD)	4
SHARK-score (T=0.95)	5
SHARK-score (T=0.9)	7
SHARK-score (T=0.85)	10
Similarity Ratio (Simratio)	4
Euclidean	10

Figure S7. Area of non-sensitivity in precision-recall curve for the alignable-disorder dataset. Area calculated via trapezium rule between the final recall point (recall=1.0) and the previous recall point for various alignment-free algorithms (a,b). This is to identify the range in which the scores are unable to distinguish between sequences in the same PFAM family (true homologs) and unrelated sequences because the maximum distance value is reached. Sequence comparison algorithms at *k*-mer lengths that have non-sensitivity area >0.003 (threshold, dotted blue line) are not considered in feature selection nor have auPRC calculated since the auPRC would be overestimated, the maximum *k*-mer length considered is summarized in c).

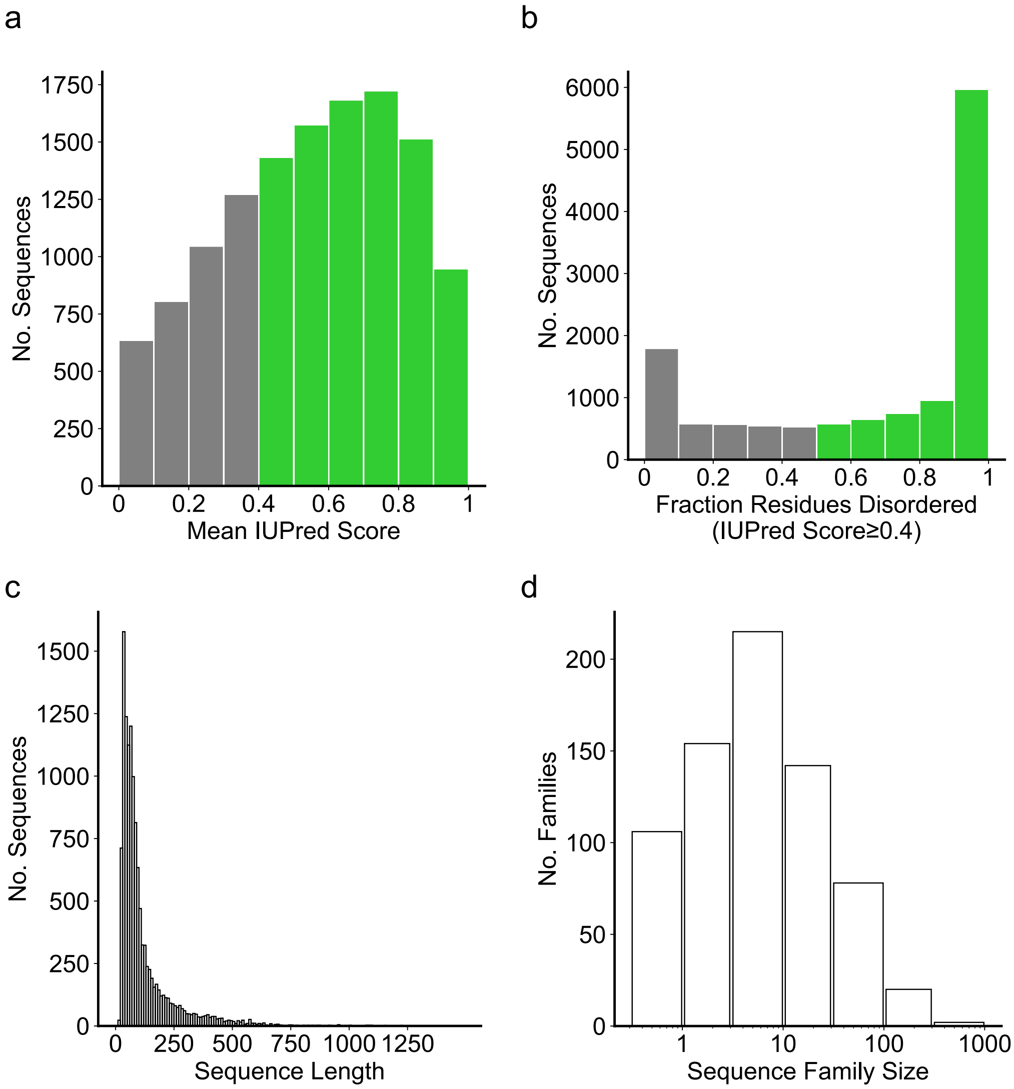

Figure S8. The unalignable orthologs dataset is enriched in sequences with disordered regions of varying lengths and sequence family sizes. a. Mean disorder of each sequence in the dataset, with a mean of 0.55 and median of 0.57, despite having no explicit disorder requirement for these non-domain, unalignable sequence. 8857/12602 (70.3%) sequences are considered disordered on average. b. Similarly, 8769/12602 (69.6%) of sequences contain extensive regions of disorder where >50% of residues are predicted to be disordered. c. Sequences are of varying lengths ranging from 15 to 1466 residues long with a mean length of 119. d. Sequence families are of various sizes after ≤50% identity filtering.

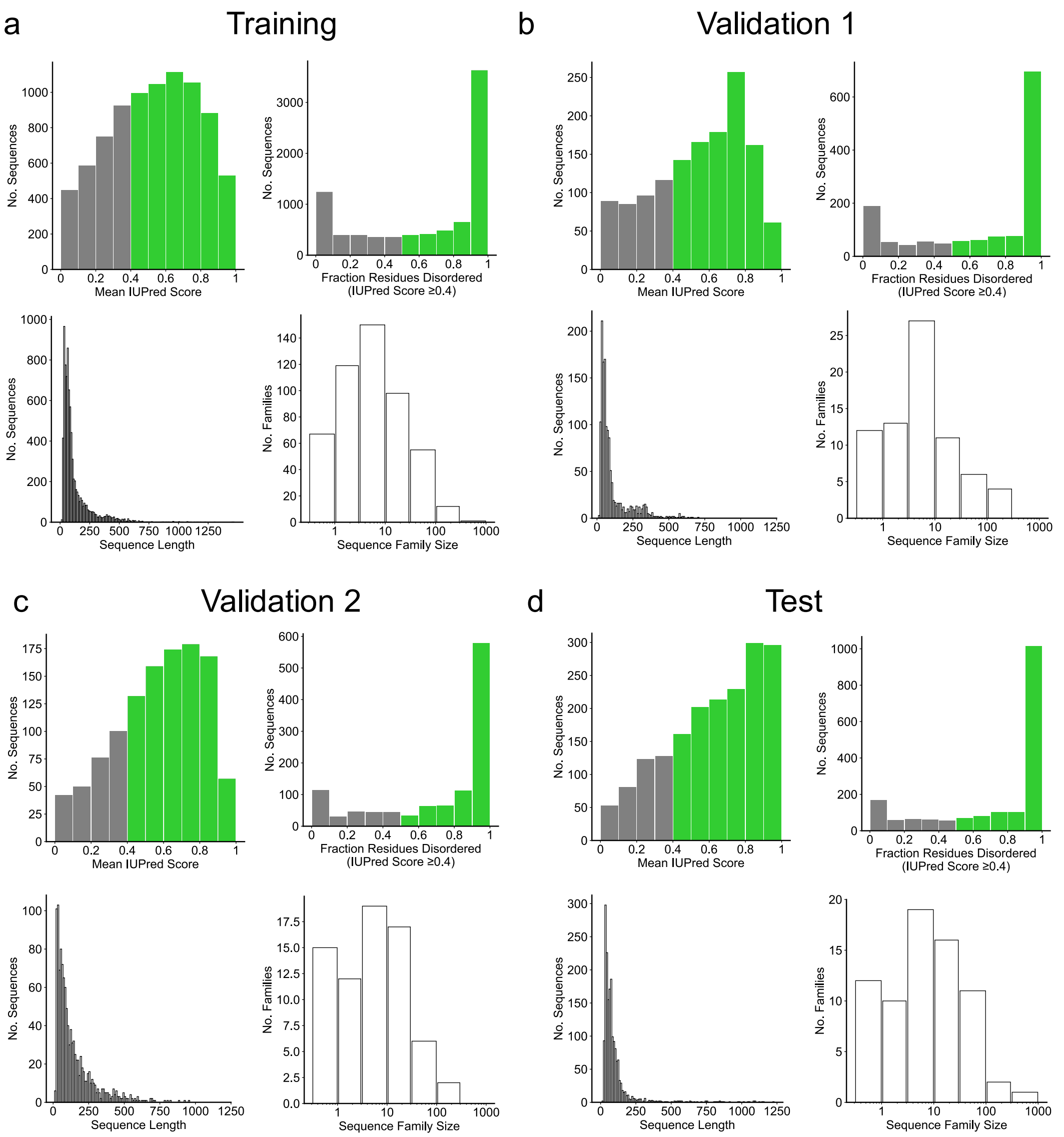

e

Dataset	Sequences (% of total)	Sequence Families	Homologous Pairs (TP)
Training	8327 (66.1)	502	709065
Validation 1	1353 (10.7)	73	131031
Validation 2	1137 (9.0)	71	92947
Test	1785 (14.2)	71	322581

Figure S9. Disordered sequences are enriched in the unalignable-ortholog sequence datasets. For each sequence subset- training (a), validation1 (b), validation2 (c) and test (d):

Top left: Distribution of mean sequence disorder, which contains 68%, 71%, 76% and 78% sequences with mean disorder ≥0.4 respectively (shown in green).

Top right: Each dataset contains sequences with extensive regions of disorder where >50% of residues are predicted to be disordered (shown in green).

Bottom right: Each dataset contains sequence families of various sizes.

Bottom left: Sequence length variation is represented across all Disprot sequence subsets.

e. Summary statistics of each unalignable-ortholog sequence dataset.

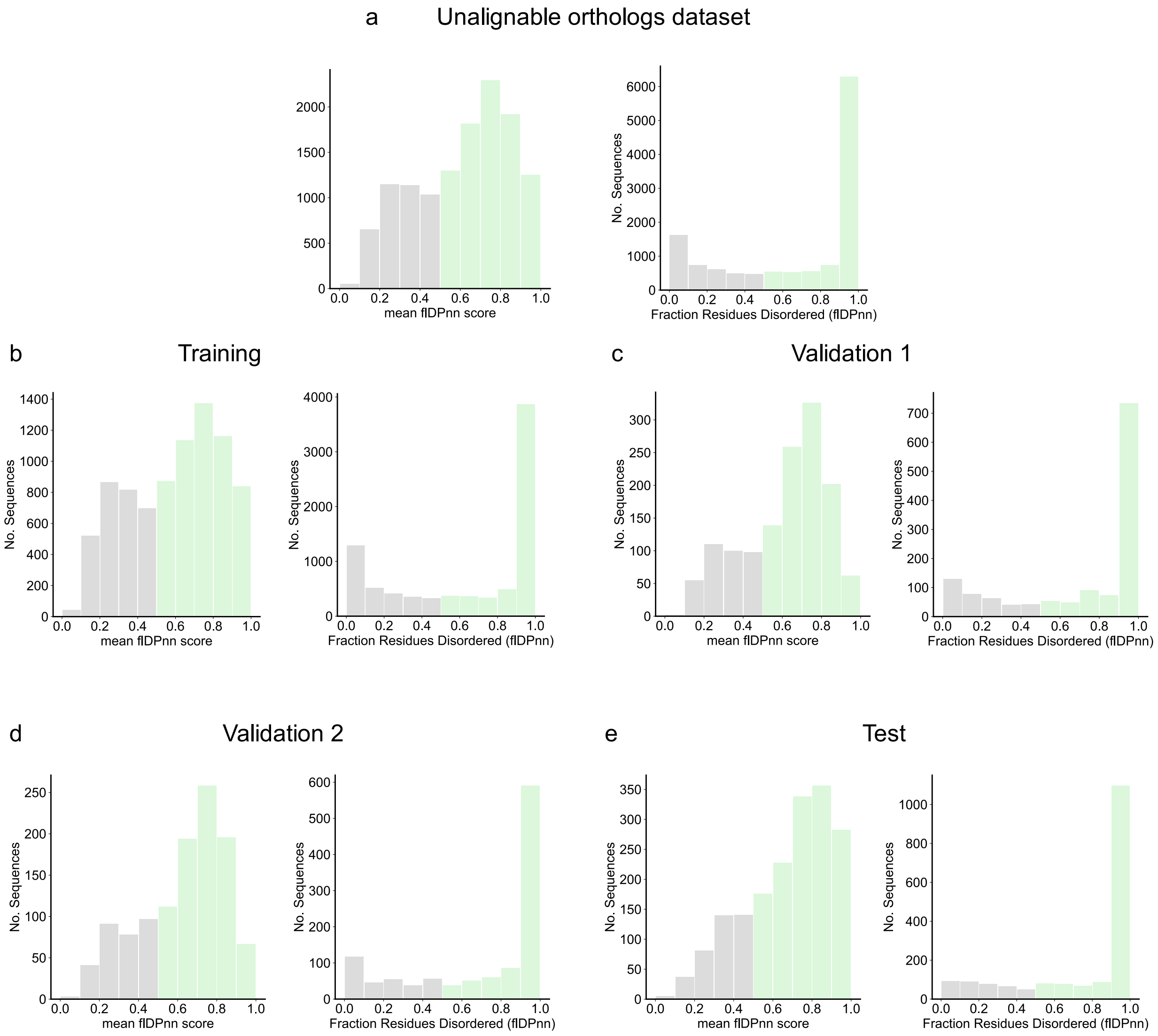

Figure S10. flDPnn-predicted disordered sequences are enriched in all unalignable-ortholog sequence datasets. Mean sequence disorder (mean flDPnn score, left) and fraction of disordered residues in each sequence (right) as predicted by flDPnn, similar to Fig. S8-S9. According to flDPnn, 68% of the unalignable-ortholog sequences have mean sequence disorder ≥0.5 (a). Disorder is retained in all splits including training (b, 65% sequences with mean sequence disorder ≥0.5), validation 1 (c, 73%), validation 2 (d, 73%) or test (e, 77%) datasets, consistent with IUPred predictions.

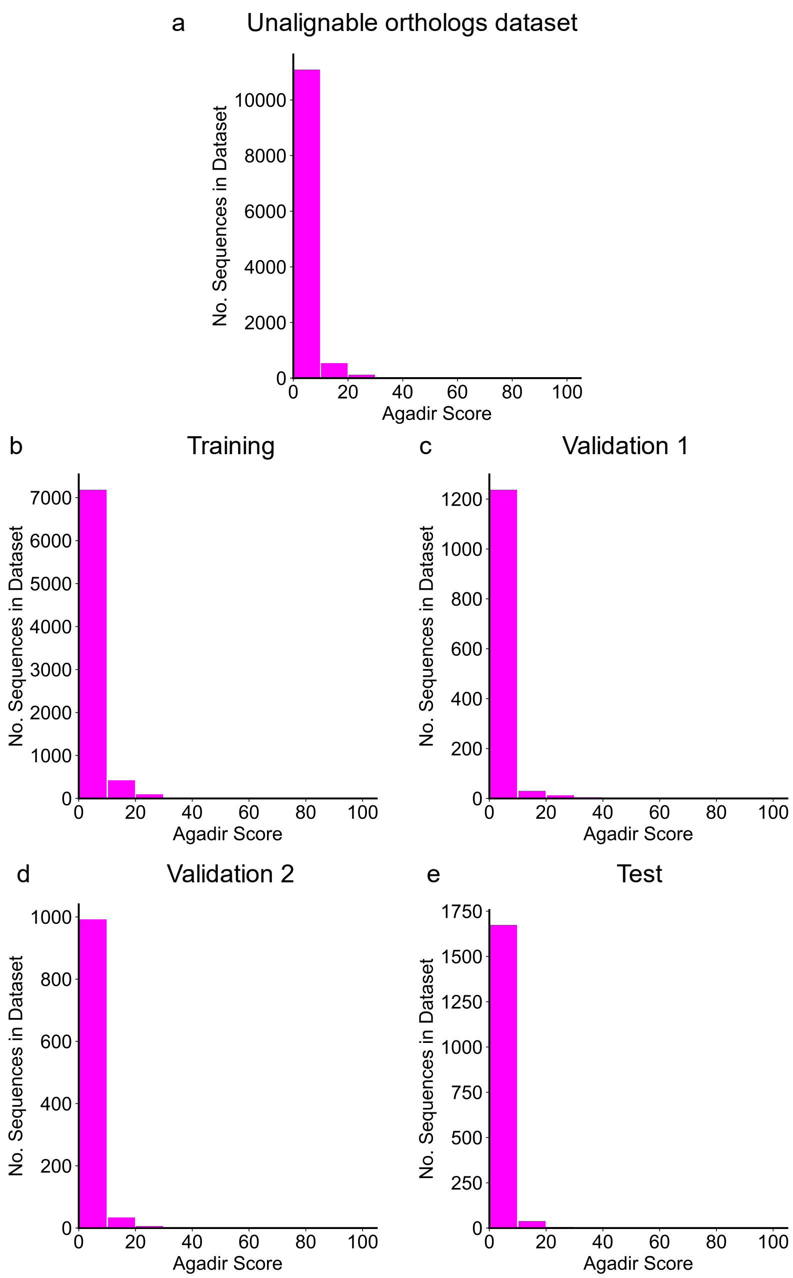

f

	Dataset				
**% sequences with helix content above**	**Full**	**Training**	**Validation 1**	**Validation 2**	**Test**
**5%**	15.57	18.60	9.80	11.83	8.44
**10%**	5.83	7.15	3.81	3.88	2.56
**20%**	1.35	1.67	1.40	0.68	0.29
**30%**	0.32	0.39	0.47	0.10	0.06

Figure S11. The unalignable orthologs dataset does not contain significant helicity. Agadir analysis shows that most sequences do not contain significant helicity. The Agadir score reflects the percentage helicity content of the sequence analyzed; the distribution is shown for the full dataset (a) as well as each split (b-e). f. Summary table of the percentage of sequences with helicity content above 5/10/20/30%. Only around 1% of sequences have >20% helicity content.

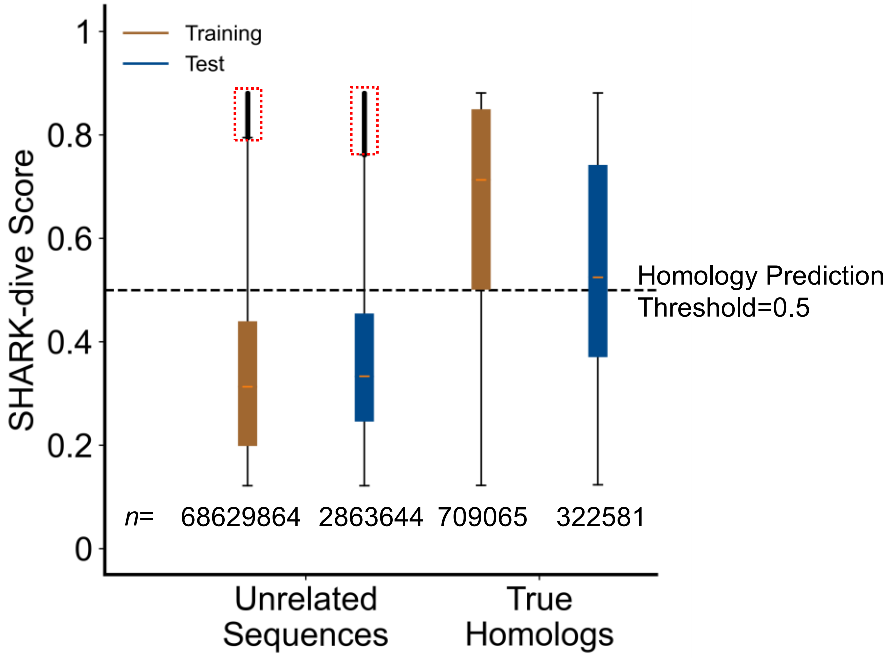

Figure S12. SHARK-dive distinguishes between true homologs and unrelated sequences in unalignable ortholog sequences. Boxplot distribution of SHARK-scores on the unalignable orthologs training and test sequences. Boxplots show median (orange line) and quartiles, outliers are shown as 'x' and boxed in red due to the overlapping points. The discriminatory power on the withheld test dataset indicates the model had not been overfitted. Orange lines in the boxes indicate the median, where in both datasets the median of unrelated sequences is below the threshold (0.5) whereas the median of the true homologs lies above the threshold.

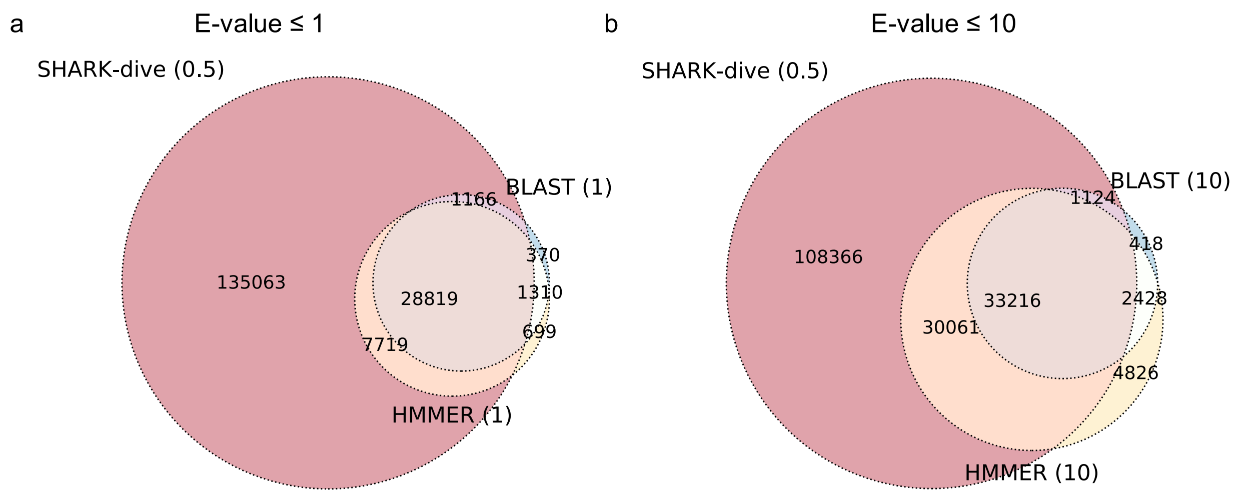

Figure S13. SHARK-dive uniquely identifies a significant fraction of true homologs in the unalignable orthologs test sequences. The number of true positives identified by BLAST, HMMER and SHARK-dive at E-value thresholds (in parenthesis) of 1 (a) and 10 (b). The Venn diagram highlights the low sensitivity of BLAST and HMMER, whereas SHARK-dive can detect a far greater unique set of true homologs.

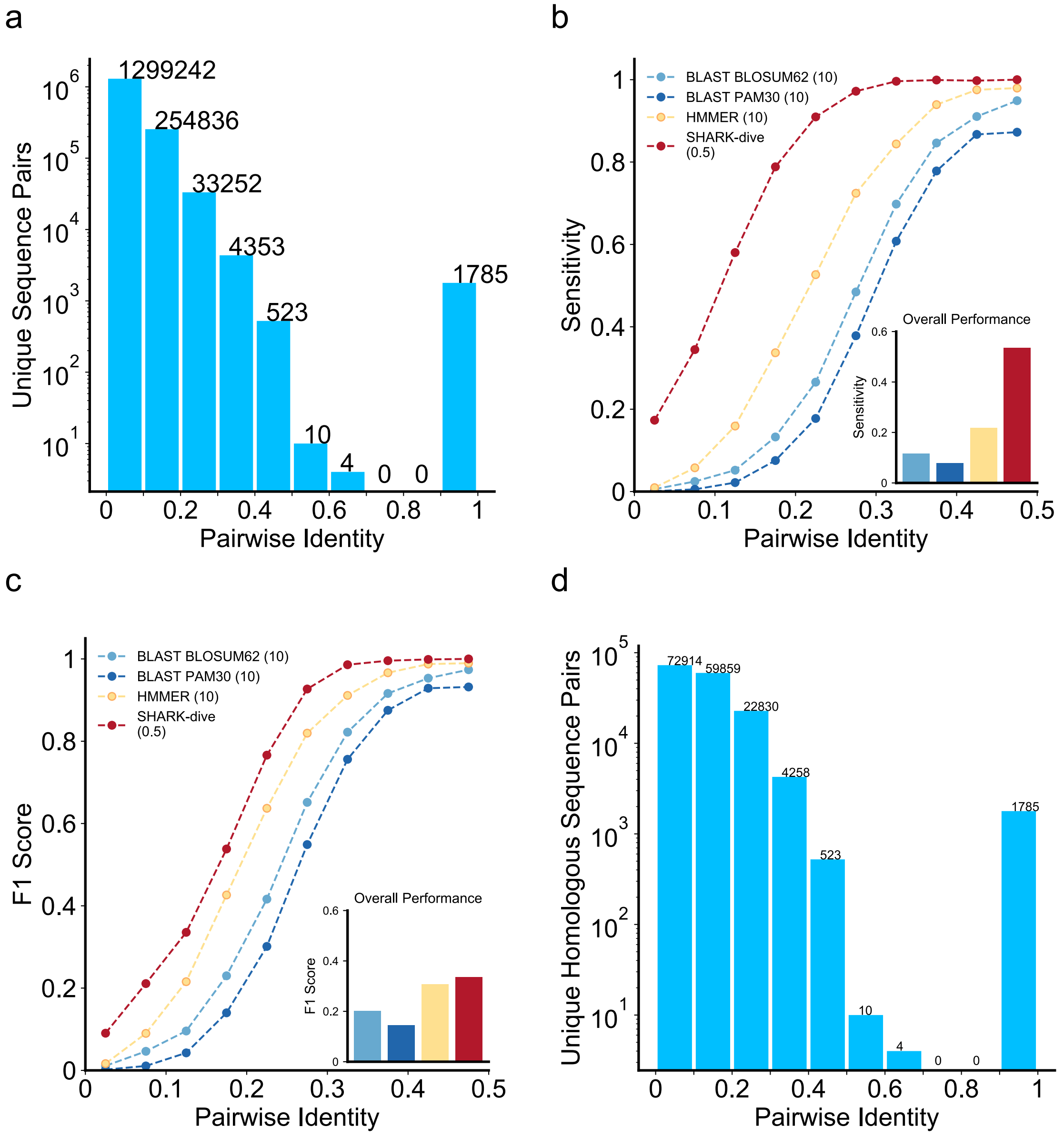

Figure S14. SHARK-dive achieves high sensitivity to remote, unalignable homologs. a. The unalignable-ortholog test sequence pairs share low identity. Pairwise identity (PID) in the unalignable orthologs test set after CD-HIT filtering. PID calculated as number of identical amino acids in alignment divided by alignment length (performed using Needleman-Wunsch with BLOSUM62 matrix with default gap penalties (see Methods). Sensitivity (b) and F1-score (c) performance of SHARK-dive and other homology assessment tools on the test dataset, stratified by PID, with the homology detection threshold shown in brackets. SHARK-dive achieves highest sensitivity and F1 across most PID bins, particularly for low identity sequence pairs. Conventional alignment-based tools such as BLAST and HMMER perform poorly in detecting remote unalignable homologs, even with a highly lenient E-value threshold of 10. (d) Most of the homologous sequence pairs in the unalignable-ortholog test dataset also share low sequence identity.

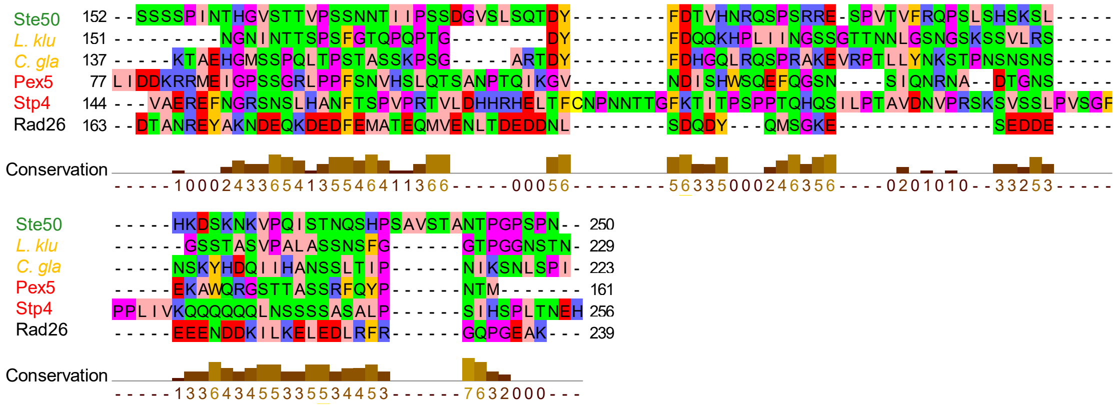

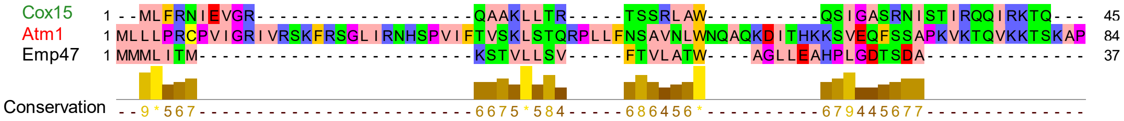

Figure S15. IDRs with low sequence conservation and poor alignment quality can be functionally homologous. Multiple Sequence Alignments of Ste50 and Cox15 with their replaced IDRs show poor alignment as indicated by the number of gaps, and the overall low conservation value (as reported in JalView).

S. cerevisiae Ste50 IDR against *L. kluyveri* Ste50 IDR

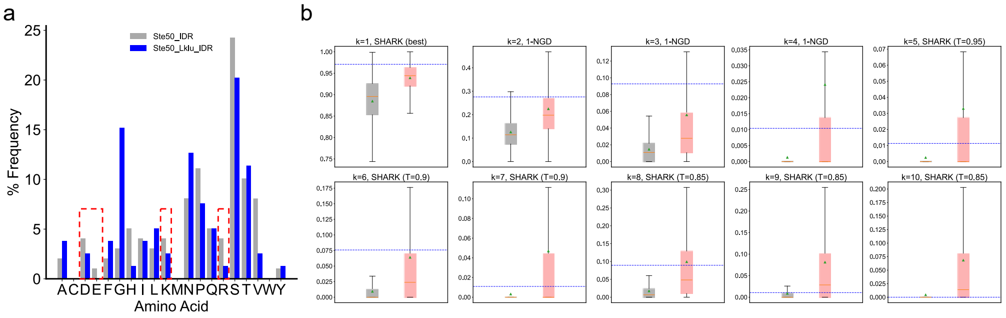

S. cerevisiae Ste50 IDR against *C. glabrata* Ste50 IDR

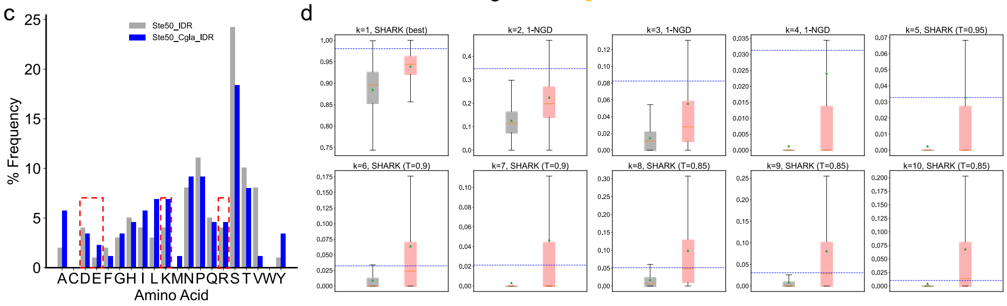

S. cerevisiae Ste50 IDR against *S. cerevisiae* Stp4 IDR

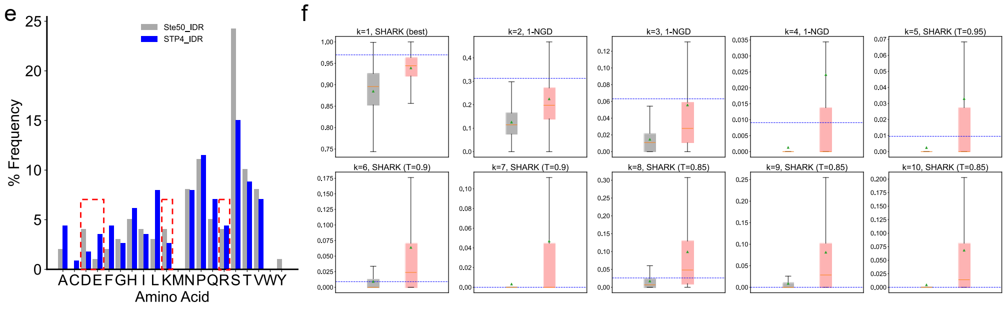

S. cerevisiae Ste50 IDR against *S. cerevisiae* Pex5 IDR

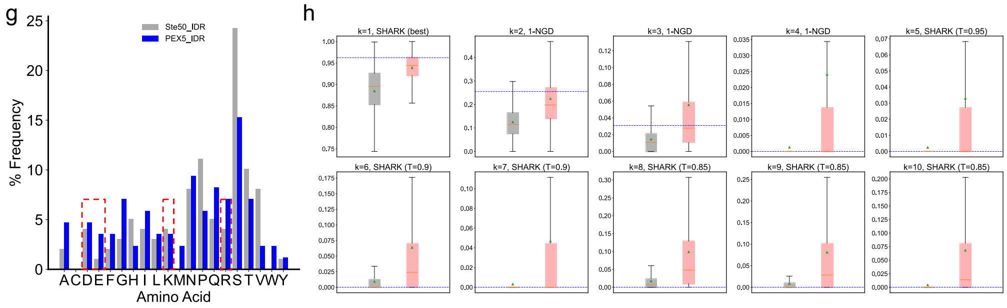

S. cerevisiae Ste50 IDR against *S. cerevisiae* Rad26 IDR

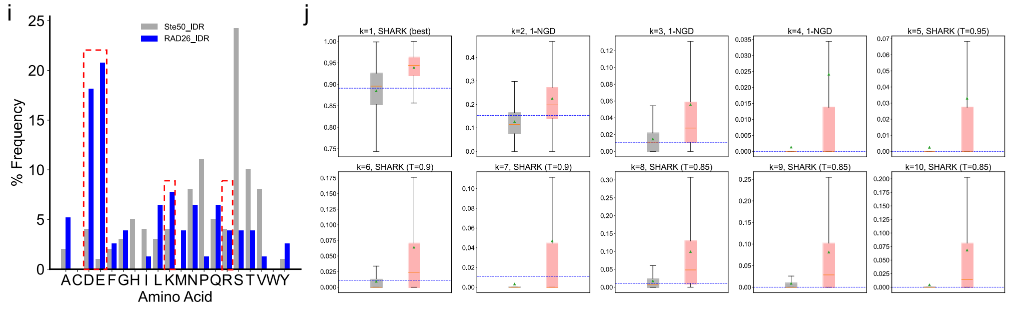

Figure S16. IDR sequences that are functionally homologous to *S. cerevisiae* Ste50 IDR share higher compositional and region similarity and is reflected in their *k*-mer scores. Amino acid compositions of evolutionary-related orthologous IDRs (*L. klu and C. gla*, highlighted in yellow) and functional analogs (S. cerevisiae Stp4 and Pex5 IDRs) are more similar to Ste50 (a, c, e, g) than the functionally non-replacing Rad26 IDR (i), which contains significantly higher proportion of negatively charged glutamate and aspartate residues, resulting in a much higher basal net charge which may disrupt function. This is reflected in the lowered 1-mer score for Rad26 (j) than the functional homologs (b, d, f, h), although other *k*-mers may also contribute to functional homology. *k*-mer scores are shown in dashed blue lines, whereas the grey boxplot represents the distribution for unrelated sequences in the SHARK-dive training set, whereas the red represents true homologs.

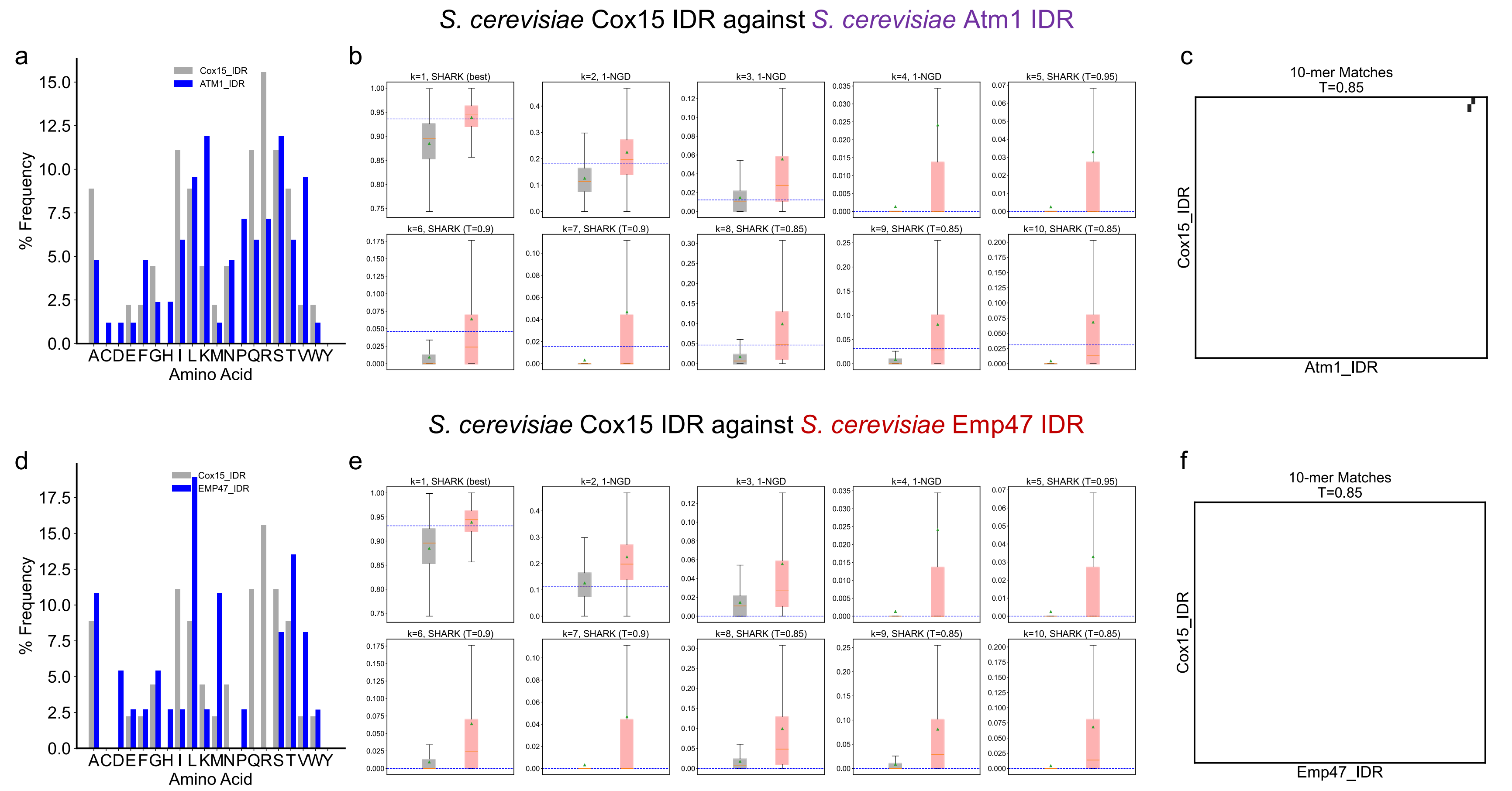

Figure S17. The functionally-homologous *S. cerevisiae* Atm1 IDR shares similar regions to the *S. cerevisiae* Cox15 IDR and is reflected in their *k*-mer scores. (a,d) Despite an overall compositional similarities between Cox15, Atm1 and Emp47 (the depletion in slightly basic Q and R residues may be partially rescued by lysine and histidines in the Emp47 IDR), Atm1 shares a similar region to Cox15 which contains multiple basic residues (c), whereas the Emp47 IDR lacks such a basic stretch (f). This may underlie the ability for the Atm1 IDR to confer mitochondrial localization whereas Emp47 cannot. (b, e) *k*-mer scores (dashed blue lines) for Atm1 and Emp47. The grey boxplot represents the distribution for unrelated sequences in the SHARK-dive training set, whereas the red represents true homologs.

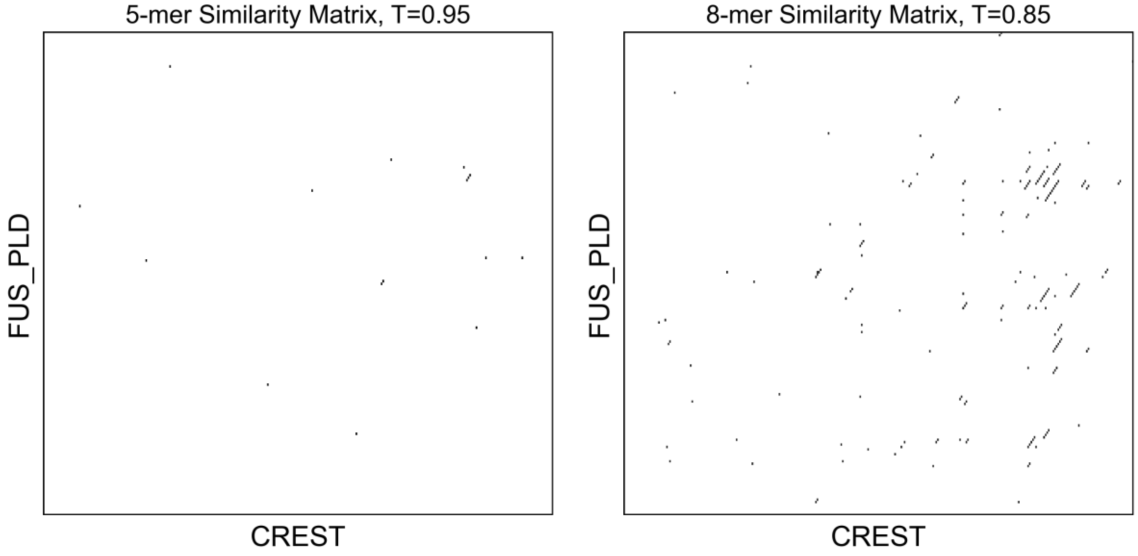

Figure S18. FUS PLD shares multiple similar *k*-mers with CREST IDR. Dot plot of similar *k*-mers (k=5, T=0.95; k=8, T=0.85) between FUS PLD and CREST (shown as black tiles). These can be mapped back onto each sequence their correspondence visualized (Fig. 5e) to identify regions of similarity between both sequences. Since the decomposition into *k*-mers removes collinear constraints, similar regions across different parts of the IDRs in different positions can be identified.

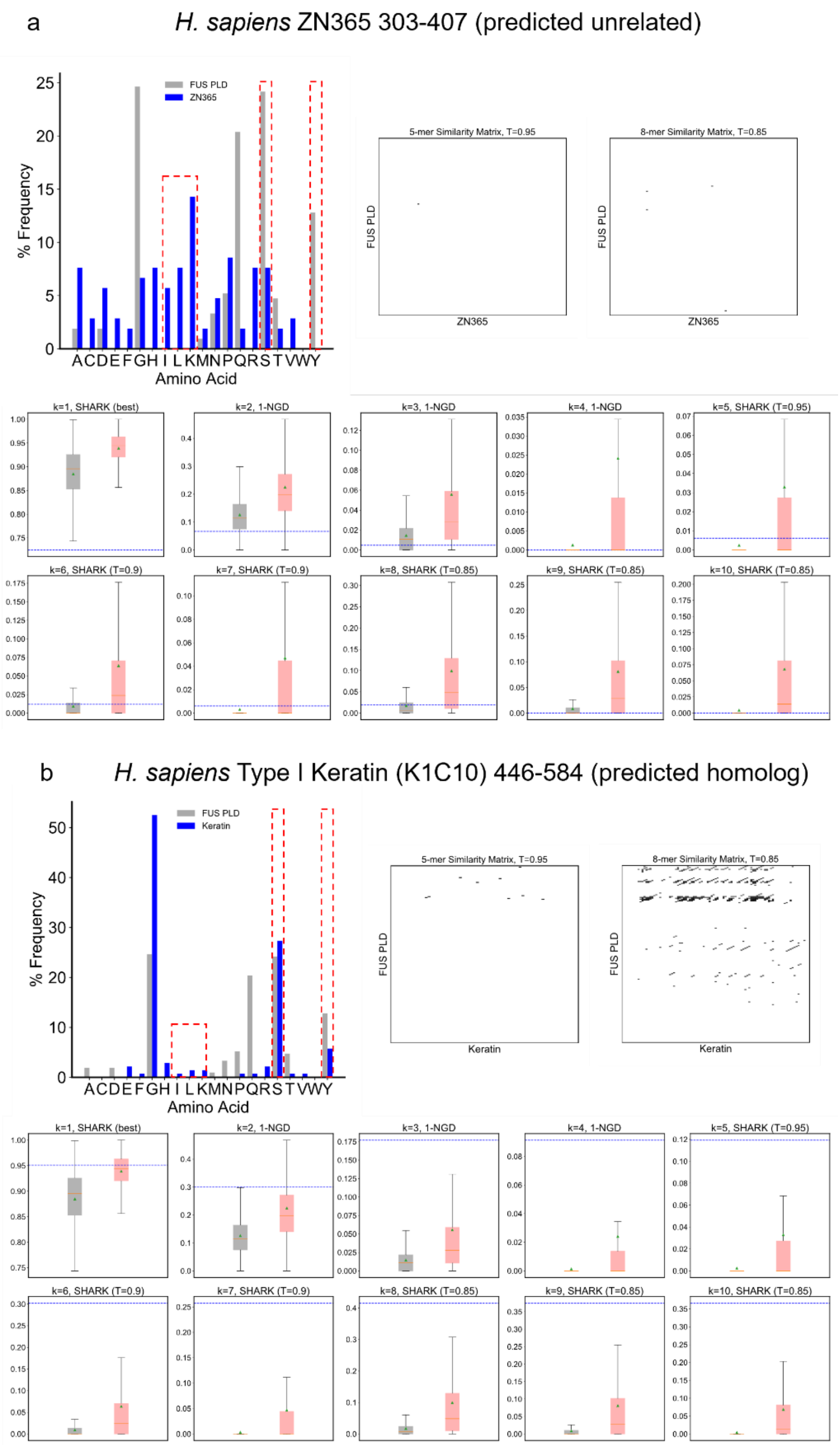

Figure S19. Predicted homologs and predicted unrelated sequences are highly dissimilar in sequence properties. FUS PLD (1-211) is searched against a database of IDRs across various proteomes. Shown here is an example of a predicted unrelated sequence (ZN365 IDR, a), as well as a predicted homolog (a C-terminal IDR of K1C10, b). For each IDR the amino acid composition is plotted against that of FUS PLD. K1C10 shows similar amino acid compositions to FUS, particularly in terms of tyrosine, serine abundance as well as depletion of aliphatics such as

leucine and isoleucine, and a lack of lysines. On the contrary, ZN365 has high abundance of K, I and L whilst containing fewer serines and no tyrosine. A similar trend is reflected in the similarity matrices where there are more highly similar, matched sequences to FUS PLD for the predicted homologs than ZN365. Altogether, this is reflected by the difference in *k*-mer scores between the two sequences (see boxplots), where K1C10 *k*-mer scores are higher than most predicted homologs whilst ZN365 *k*-mer scores are closer to the distribution for unrelated sequences. Accordingly, K1C10 is predicted to be homologous to the FUS PLD whereas ZN365 is not. For *k*=2-4, NGD scores are visualized as 1-NGD (otherwise known as the Normalized Google Similarity) such that higher scores represent higher similarity for ease of interpretation. Boxplots show median (orange line), mean (green triangle) and quartiles. Outliers are not shown but can be found in Fig. S23.

FUS PLD against *H. sapiens* RALY 202-306 (predicted homolog)

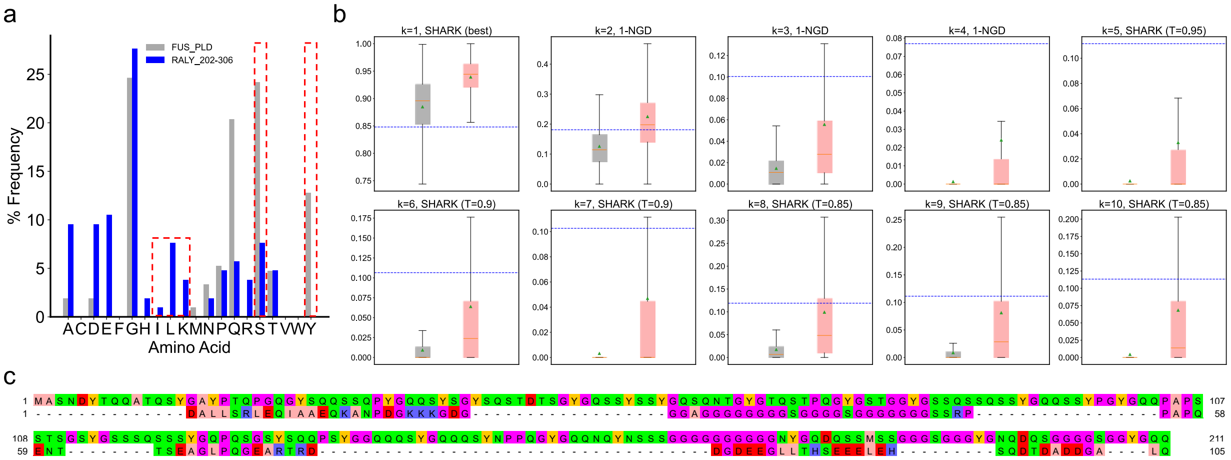

Figure S20. SHARK-dive predicts FUS PLD-RALY IDR homology despite low amino acid compositional similarity. Despite a dissimilar composition (a), SHARK-dive nonetheless predicts homology between the FUS PLD an IDR in the human RALY protein, which shares similar *k*-mers leading to high *k*-mer scores (particularly for *k*=4 and 5, b) despite poor alignment (9.5% identity, c).

FUS RBD against *H. sapiens* RALY 202-306 (predicted homolog)

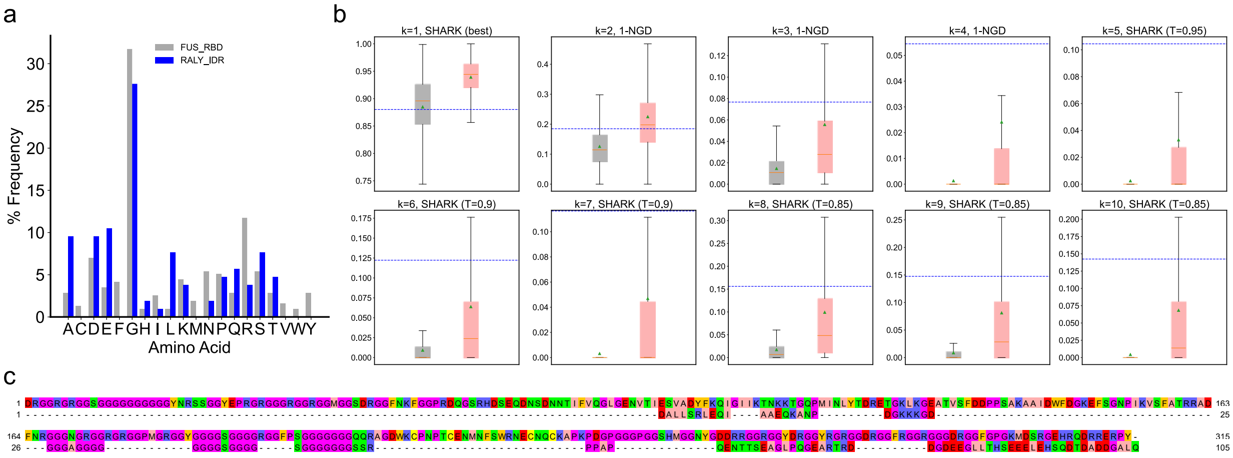

Figure S21. SHARK-dive predicts FUS RBD-RALY IDR homology despite low amino acid compositional similarity. Despite a dissimilar composition (a), SHARK-dive nonetheless predicts homology between the FUS RBD and an IDR in the human RALY protein, which shares similar *k*-mers leading to high *k*-mer scores (particularly for *k*=4 and 5, b) despite poor alignment.

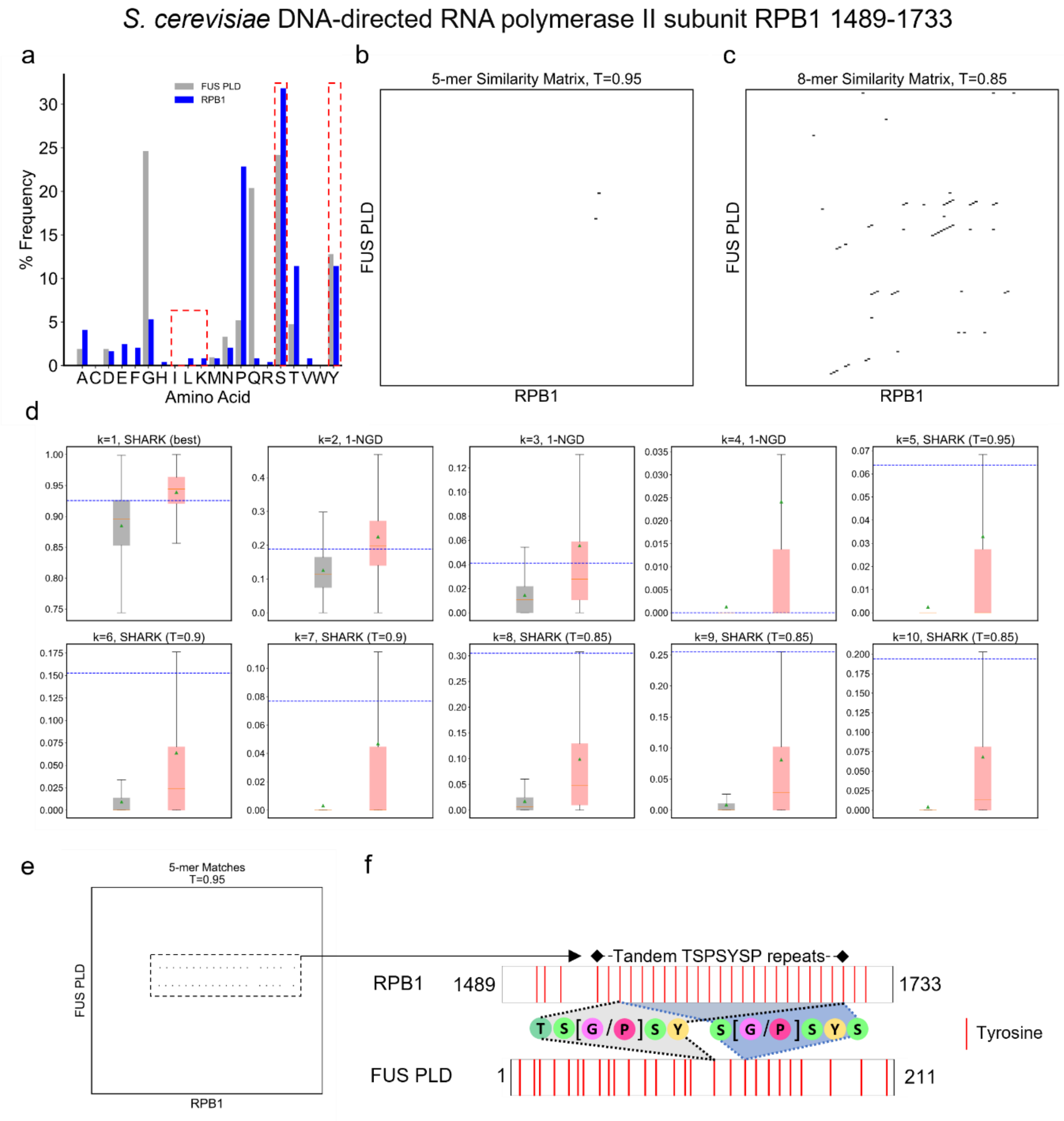

Figure S22. SHARK-dive predicts a yeast IDR to be homologous to human FUS PLD. SHARK-dive can also be used to predict homologous IDRs in other species, such as the RPB1 IDR in *S. cerevisiae*, where there are no known orthologs but various pathways where FUS is involved are shared between the two species(29, 30). Compared to the CREST IDR (see Fig. 5 c,d), RPB1 shares lower compositional similarities to FUS PLD, as reflected by the lower compositional (*k*=1) score (d). Nonetheless, it does share multiple similar *k*-mers to FUS PLD (*k*=5 and *k*=8 shown in panels b and c respectively). Notably, mapping the highly similar 5-mers (black dots in b) back onto their respective sequences (e), reveals that the 5-mer matches along the sequences map onto a highly repetitive and functional region in RPB1 (f) which would be difficult to align, which contributes to its higher 5-mer score. Whilst only *k=5,* T=0.95 and *k*=8, *T*=0.85 similarity matrices are shown, others can be generated by using the Jupyter Notebook (https://git.mpi-cbg.de/tothpetroczylab/shark/-/tree/1-version-release-workflow/notebooks).
Note: In panel d, for *k*=2-4, NGD scores are visualized as 1-NGD (otherwise known as the Normalized Google Similarity) such that higher scores represent higher similarity for ease of interpretation. Boxplots show median (orange line), mean (green triangle) and quartiles, outliers are shown as ‘x’.

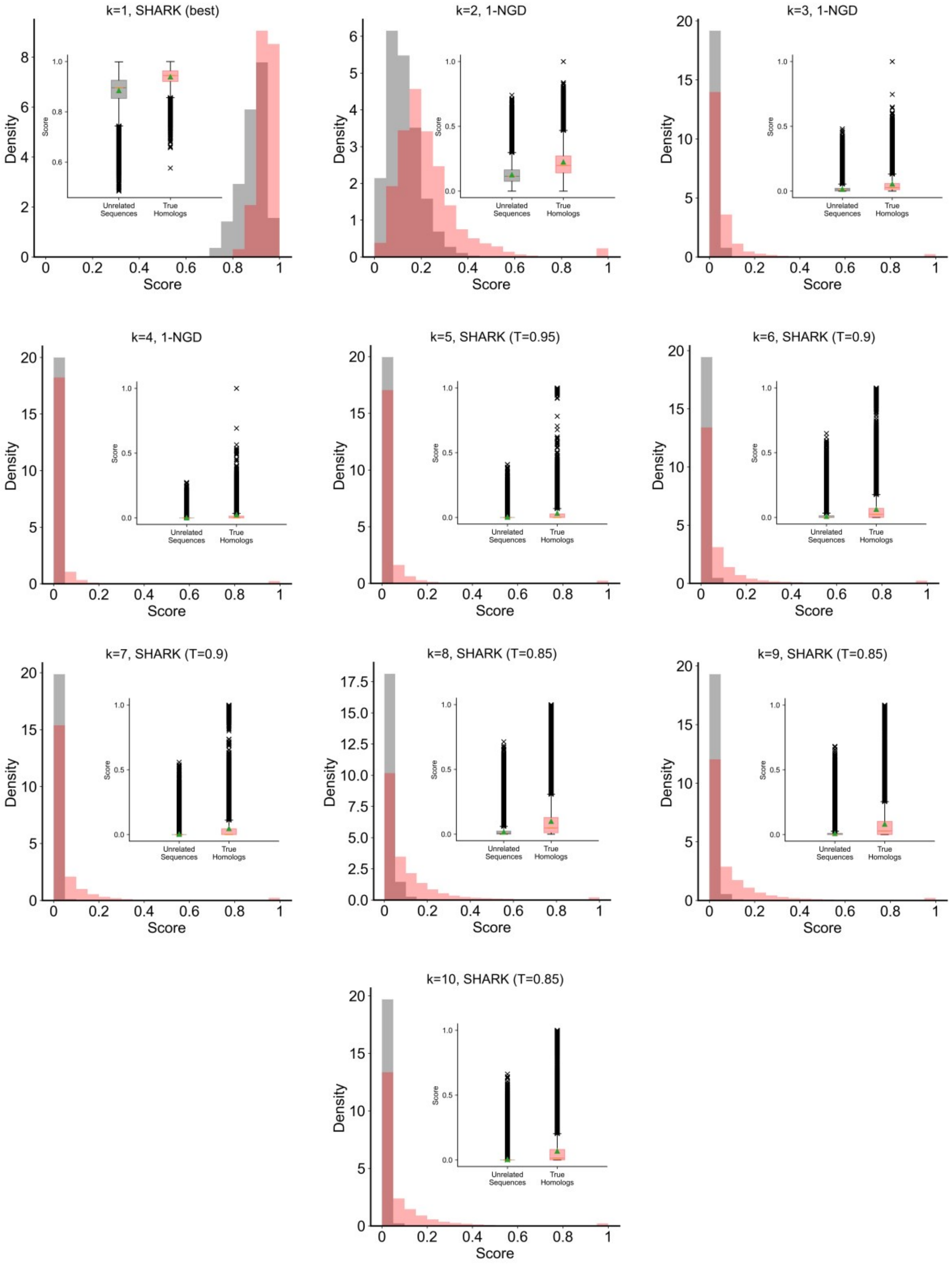

Figure S23. Individual SHARK-dive features provide discriminatory power over true homologs and unrelated sequences. Histogram distributions in the unalignable orthologs training set between true homologs and unrelated sequences, with boxplot distributions shown in inset (showing median (orange line), mean (green triangle) and quartiles, outliers as 'x'). Note: for *k*=2-4, NGD scores are visualized as 1-NGD (otherwise known as the Normalized Google Similarity) such that higher scores represent higher similarity for ease of interpretation.

WT IDR:	Ste50					Cox15	
Replaced IDR:	*L.klu*	*C.gla*	Pex5	Stp4	Rad26	Atm1	Emp47
Experiment (ground-truth)	✓	✓	✓	✓	✗	✓	✗
SHARK-dive	✓	✓	✓	✓	✗	✓	✗
DEDAL	✓	✓	✗	✓	✗	✗	✗
BLAST (BLOSUM62)	✗	✓	✗	✗	✗	✗	✗
HMMER	✗	✓	✗	✗	✗	✗	✗

Figure S24. Performance of DEDAL compared with other tools, related to Figure 4d. DEDAL outperforms BLAST and HMMER in detection of some IDRs capable of functional replacement but is unable to capture all true positives. SHARK-dive remains the only tool capable of identifying all IDRs capable of functional replacement in these experiments.

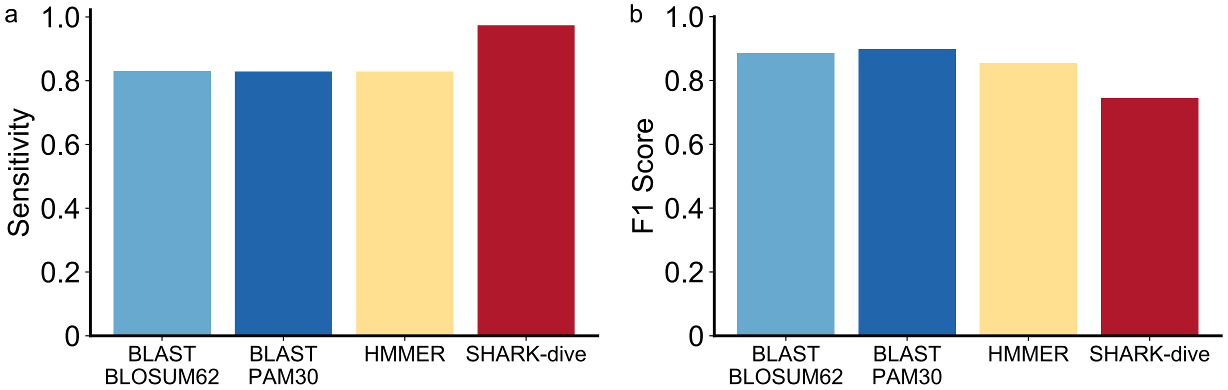

c

Method, Threshold	Sensitivity	Specificity	Precision	Accuracy	F1
BLAST short,10	0.853	0.990	0.968	0.955	**0.907**
BLAST PAM70, 10	0.831	0.996	0.985	0.953	0.902
BLAST PAM30, 10	0.829	0.995	0.981	0.952	0.899
BLAST BLOSUM80, 10	0.834	0.991	0.968	0.950	0.896
BLAST BLOSUM90, 11	0.834	0.988	0.959	0.948	0.892
BLAST PAM250, 10	0.837	0.983	0.944	0.945	0.887
BLAST BLOSUM62, 10	0.830	0.985	0.951	0.945	0.886
BLAST PAM30, 10	0.830	0.984	0.946	0.944	0.884
BLAST BLOSUM50, 10	0.829	0.981	0.939	0.942	0.880
HMMER, 10	0.829	0.962	0.883	0.927	0.855
SHARK-dive, 0.5	**0.974**	0.775	0.602	0.827	0.745

Fig S25. SHARK-dive offers superior sensitivity to domain-less unalignable proteins albeit at the cost of overall performance. We note that this is a small dataset containing 52 full-length sequences across 5 sequence families in the unalignable orthologs test dataset that have no Pfam domain annotations. Sensitivity (a) and overall (F1 score, b) performance in detecting homology. SHARK-dive achieves highest sensitivity but predicts more false positives, leading to inferior overall performance, as summarized in the final table (c, where shaded rows indicate the homology detection tools shown in a and b).

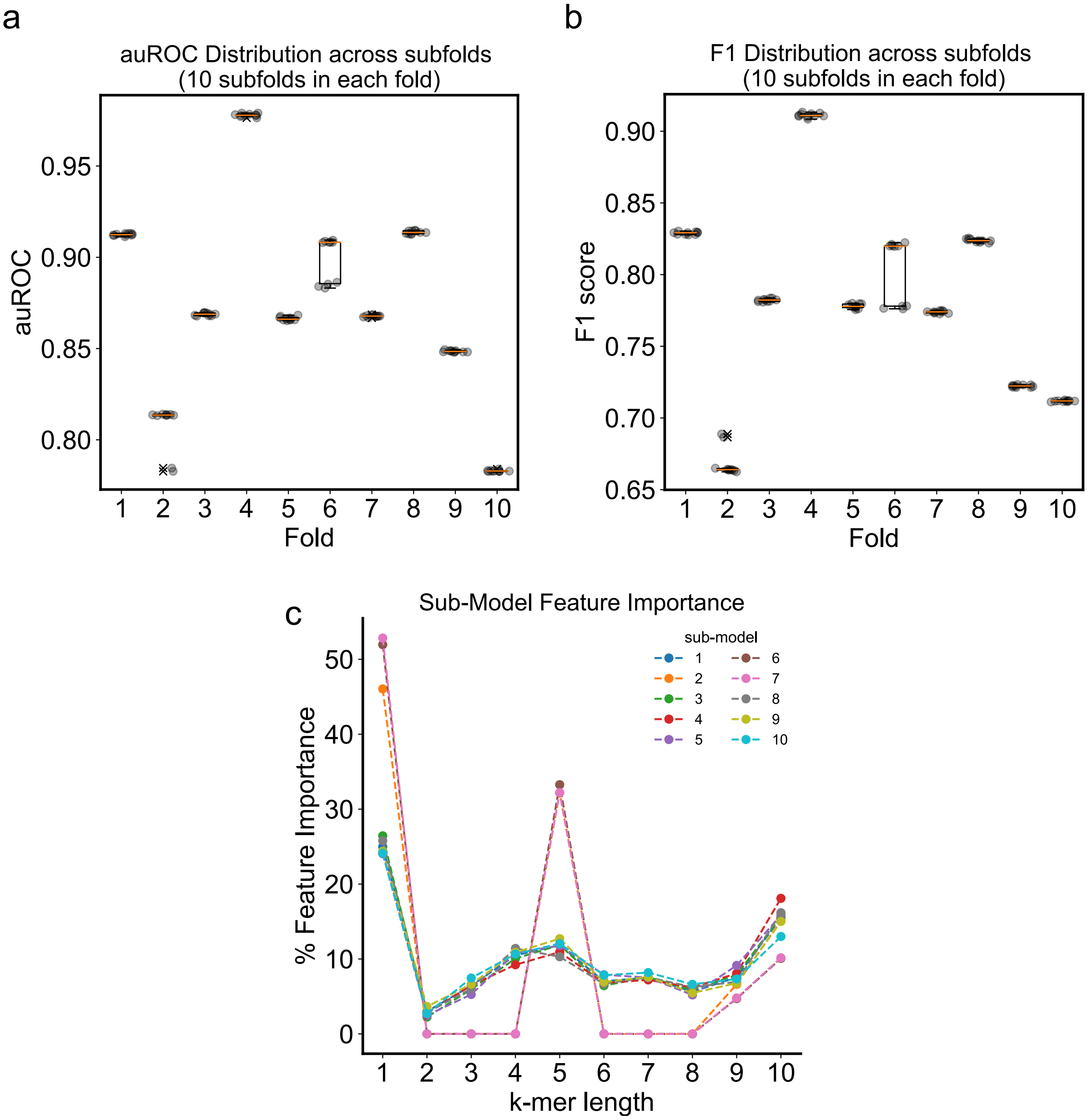

Figure S26. Performance and feature importance are consistent across sub-folds and sub-models. Since the number of unrelated sequences (true negatives, TN) far exceeds the number of true homologs (true positives, TP), we trained 10 sub-folds for each sub-model. Each sub-fold contains a training and validation set: the set of true homologs are identical across the sub-folds but the set of unrelated sequences is different. To assess the impact of different unrelated sequences on model training, the area under the receiver operating characteristic curve (auROC, a) and F1 score performance (b) of each sub-fold model on its respective validation dataset was calculated. Since the performance is largely similar, we concluded that the choice of unrelated sequence pairs does not matter significantly (<0.05 difference F1/auROC between min and max). Nonetheless, for each fold we chose the best sub-fold on the validation1 early-stopping dataset used to prevent over-fitting, this is known as a sub-model (i.e. 10 sub-models altogether form the SHARK-dive model). Boxplots show median (orange line) and quartiles, outliers are shown as 'x'. Individual auROC and F1 metrics are shown as grey circles c. Feature importance of each sub-model shows similar trends, with *k*=1, 5 and 10 being the most important in all sub-models.

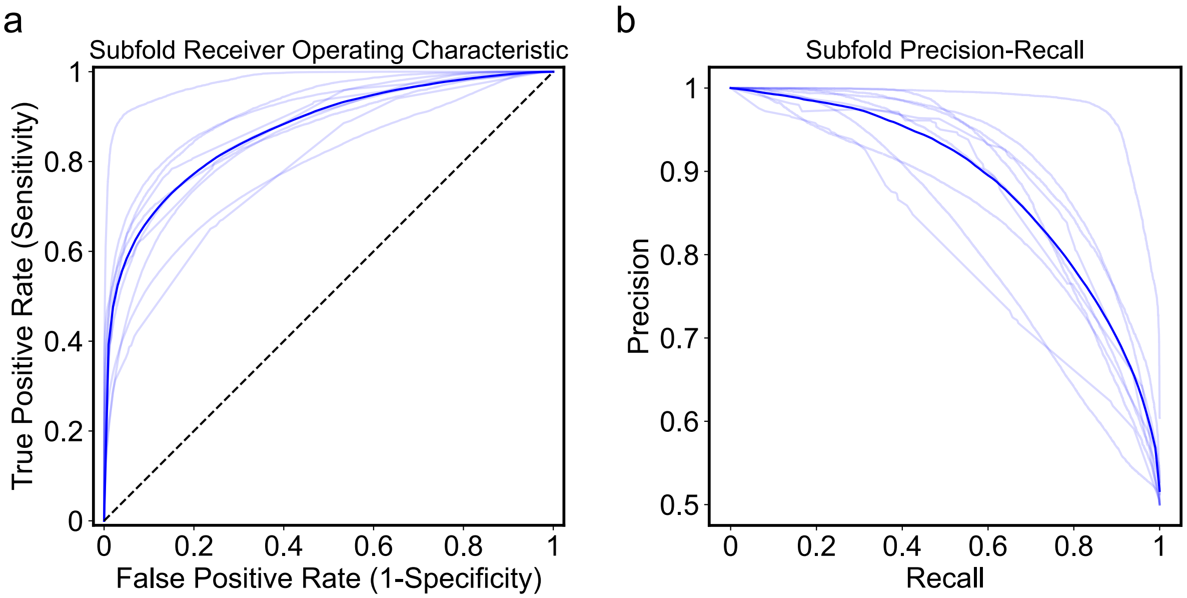

Figure S27. Ensemble sub-models show consistent performance on validation datasets. Receiver Operating Characteristic (a) and Precision-Recall (b) curves for each sub-model on its validation dataset. Each ensemble sub-model has a specific validation dataset with withheld sequences not used during model training to assess the performance of the model. Despite variations in sub-model performance, they all show superior performance to a random classifier (dotted line, ROC plot). Differences are due to differences in number of sequences used because sequence family sizes are different, but each dataset is nonetheless balanced in the number of true homologs (TP) and unrelated sequences (TN). There are between 451-453 training families and 49-51 validation families in each fold.

a

Runtime

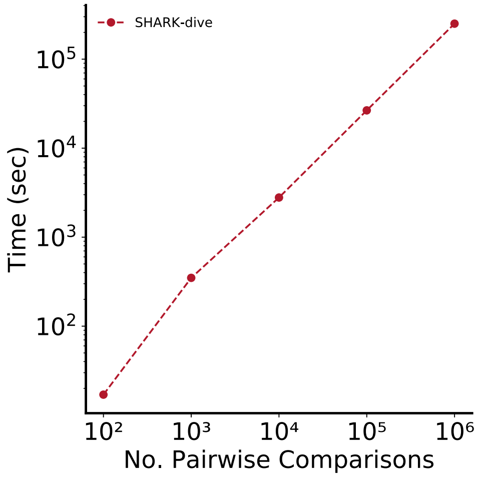

No. Pairwise Comparisons	Time taken for SHARK-dive (sec)
100	17
1000	349
10000	2789
100000	26614
1000000	250798 (~70 h)

b

Memory

No. Pairwise Comparisons	SHARK-dive Memory Usage (GB)
100	0.9
1000000	1.4

Figure S28. Runtime and memory analysis. SHARK-dive runtime scales linearly in a simulated database search for a protein sequence while maintaining low memory requirements. For further information, see Methods (Runtime and memory analysis).

Supplementary Tables

Table S1. Parameters used for benchmarking Smith-Waterman local alignment

<table>
<tr><th>Substitution Matrix</th><th>Gap Opening Penalty (G) (31)</th><th>Gap Extension Penalty (L)</th><th>Notes</th><th>Ref</th></tr>
<tr><td>BLOSUM62</td><td>-11</td><td>-1</td><td>BLASTp default parameters</td><td rowspan="2">(32–34)</td></tr>
<tr><td>PAM30</td><td>-9</td><td>-1</td><td>BLASTp-short default parameters</td></tr>
<tr><td>PFASUM70</td><td>-15</td><td>-1.5</td><td>generated using DECIPHER(35, 36) package in R and rounded to nearest integer as per Keul et al.(18)</td><td>(18)</td></tr>
<tr><td>EDSSMat60</td><td>-6</td><td>-2</td><td rowspan="7">Medium Disorder (MD)</td><td rowspan="14">(37)</td></tr>
<tr><td>EDSSMat80</td><td>-5</td><td>-2</td></tr>
<tr><td>EDSSMat50</td><td>-6</td><td>-2</td></tr>
<tr><td>EDSSMat90</td><td>-5</td><td>-2</td></tr>
<tr><td>EDSSMat62</td><td>-5</td><td>-2</td></tr>
<tr><td>EDSSMat70</td><td>-5</td><td>-2</td></tr>
<tr><td>EDSSMat75</td><td>-5</td><td>-2</td></tr>
<tr><td>EDSSMat60</td><td>-14</td><td>-3</td><td rowspan="7">High Disorder (HD)</td></tr>
<tr><td>EDSSMat80</td><td>-15</td><td>-3</td></tr>
<tr><td>EDSSMat50</td><td>-18</td><td>-2</td></tr>
<tr><td>EDSSMat90</td><td>-19</td><td>-2</td></tr>
<tr><td>EDSSMat62</td><td>-19</td><td>-2</td></tr>
<tr><td>EDSSMat70</td><td>-19</td><td>-2</td></tr>
<tr><td>EDSSMat75</td><td>-19</td><td>-2</td></tr>
</table>

Note: Matrices shaded in grey were also used in benchmarking the alignable-disorder dataset

Table S2. The best performing alignment-free algorithm selected for each *k*-mer length.

k	Algorithm
1	SHARK-score (best)
2	NGD
3	NGD
4	NGD
5	SHARK-score (T=0.95)
6	SHARK-score (T=0.9)
7	SHARK-score (T=0.9)
8	SHARK-score (T=0.85)
9	SHARK-score (T=0.85)
10	SHARK-score (T=0.85)

Note: NGD: Normalized Google Distance.

Table S3. Benchmarking against Local Alignment (Smith-Waterman algorithm) on unalignable-orthologs dataset

Method, Threshold	Recall	Specificity	Precision	Accuracy	f1	auPRC
SHARK-dive, 0.5	**0.536**	0.814	0.245	0.786	**0.336**	**0.344**
EDSSMat60 (MD), 35	0.198	0.956	0.335	0.879	0.249	0.258
EDSSMat80 (MD), 37	0.200	0.954	0.332	0.878	0.250	0.256
EDSSMat50 (MD), 36	0.197	0.956	0.336	0.879	0.249	0.256
EDSSMat90 (MD), 37	0.198	0.956	0.337	0.879	0.250	0.256
EDSSMat62 (MD), 37	0.202	0.953	0.326	0.877	0.250	0.256
EDSSMat70 (MD), 37	0.201	0.954	0.328	0.877	0.249	0.256
EDSSMat75 (MD), 37	0.202	0.953	0.325	0.877	0.249	0.256
EDSSMat60 (HD), 34	0.170	0.973	0.417	0.892	0.242	0.253
EDSSMat80 (HD), 34	0.173	0.972	0.407	0.891	0.242	0.253
EDSSMat62 (HD), 34	0.173	0.971	0.402	0.890	0.242	0.253
EDSSMat70 (HD), 34	0.172	0.971	0.404	0.890	0.242	0.253
EDSSMat75 (HD), 34	0.173	0.971	0.400	0.890	0.241	0.253
EDSSMat90 (HD), 34	0.171	0.973	0.412	0.891	0.241	0.252
EDSSMat50 (HD), 34	0.175	0.969	0.388	0.889	0.241	0.252
PAM30, 35	0.244	0.916	0.246	0.848	0.245	0.250
PFASUM70, 41.5	0.376	0.792	0.169	0.750	0.233	0.238
BLOSUM62, 31	0.341	0.819	0.175	0.771	0.231	0.237

Note: For completeness, specificity and accuracy values are included although we note that these metrics, much like the auROC, are not suitable for our imbalanced test dataset due to the high number of unrelated pairs from an all-vs-all comparison. Table is sorted in descending order of auPRC, with highest auPRC and F1 shown in bold. Shaded in blue are the precision-recall curves shown in Fig. 3d.

Table S4. Tools and parameters used for benchmarking against alignment-based homology search tools

Tool	Substitution Matrix	Gap Opening Penalty (*G*) (31)	Gap Extension Penalty (*L*)	Thresholds tested	Notes	Ref.
BLASTp	BLOSUM62	-11	-1	E-value: 10,1,0.05	BLASTp default parameters	(32, 38)
	PAM30	-9	-1			
	PAM30	-9	-1		BLASTp-short (no composition-based statistics, word_size=2)	
	BLOSUM50	-13	-2			
	PAM250	-14	-2			
	BLOSUM90	-10	-1			
	BLOSUM45	-15	-2			
	BLOSUM80	-10	-1			
	PAM70	-10	-1			
pHMMER	Default	Default	Default	E-value: 10,1,0.001 Bitscore: 7	--max option used for maximum sensitivity	(39, 40)

Note: For BLASTp and pHMMER reporting E-values were set arbitrarily high (10^4 for BLASTp, 10^{14} for pHMMER)

Table S5. Benchmarking on the unalignable-orthologs test dataset

Method, Threshold	Recall	Specificity	Precision	Accuracy	F1
SHARK-dive, 0.5	**0.536**	0.814	0.245	0.786	**0.336**
pHMMER E-value, 10 (default reporting E-value)	0.219	0.977	0.515	0.900	0.307
BLAST BLOSUM50, 10	0.134	0.993	0.694	0.906	0.225
BLAST PAM250, 10	0.129	0.996	0.765	0.908	0.221
pHMMER bitscore, 7 (default reporting bitscore)	0.121	0.998	0.867	0.909	0.213
pHMMER E-value, 1 (default reporting E-value on webserver)	0.119	0.998	0.856	0.909	0.210
BLAST BLOSUM90, 10	0.116	0.997	0.829	0.908	0.203
BLAST BLOSUM62, 10	0.115	0.996	0.787	0.907	0.201
BLAST BLOSUM45 10	0.111	0.996	0.740	0.906	0.193
BLAST PAM250, 1	0.106	0.999	0.954	0.909	0.191
BLAST BLOSUM50, 1	0.105	0.999	0.944	0.909	0.190
BLAST BLOSUM80, 10	0.103	0.998	0.860	0.907	0.184
BLAST short, 10	0.102	0.998	0.867	0.908	0.183
BLAST BLOSUM62, 1	0.098	1.000	0.964	0.908	0.178
BLAST BLOSUM45, 1	0.098	0.999	0.942	0.908	0.177
BLAST BLOSUM90, 1	0.095	1.000	0.974	0.908	0.173
BLAST PAM70, 10	0.094	0.999	0.917	0.907	0.171
BLAST BLOSUM80, 1	0.089	1.000	0.978	0.908	0.164
BLAST short, 1	0.084	1.000	0.980	0.907	0.155
BLAST PAM70, 1	0.083	1.000	0.988	0.907	0.153
BLAST BLOSUM45, 0.05	0.081	1.000	0.995	0.907	0.151
BLAST PAM250, 0.05	0.081	1.000	0.997	0.907	0.150
BLAST BLOSUM50, 0.05	0.081	1.000	0.996	0.907	0.149
BLAST BLOSUM62, 0.05	0.078	1.000	0.997	0.907	0.145
BLAST PAM30, 10	0.078	0.999	0.938	0.906	0.145
BLAST BLOSUM90, 0.05	0.074	1.000	0.998	0.906	0.138
BLAST BLOSUM80, 0.05	0.072	1.000	0.998	0.906	0.135
BLAST PAM30, 1	0.071	1.000	0.989	0.906	0.132
BLAST PAM70, 0.05	0.068	1.000	0.999	0.906	0.127
BLAST short, 0.05	0.066	1.000	0.998	0.905	0.124
pHMMER E-value, 0.001	0.064	1.000	0.999	0.905	0.121
BLAST PAM30, 0.05	0.060	1.000	0.999	0.905	0.113

Note: For completeness, specificity and accuracy values are included although we note that these metrics, much like the auROC, are not suitable for our imbalanced test dataset due to the high number of unrelated pairs from an all-vs-all comparison. Table is sorted in descending order of F1, with highest recall (sensitivity) and F1 shown in bold. Shaded in blue are thresholds shown in Fig. 3e and f.

Table S6. Pairwise identity of sequences compared in Fig. 4

Query	Entry	Pairwise Identity
sp|P25344|STE50_YEAST/152-250	sp|P35056|PEX5_YEAST/77-161	0.094
sp|P25344|STE50_YEAST/152-250	sp|Q07351|STP4_YEAST/144-256	0.133
sp|P25344|STE50_YEAST/152-250	sp|P40352|RAD26_YEAST/163-239	0.018
sp|P25344|STE50_YEAST/152-250	STE50_ORTHOLOG/C.gla/137-223	0.248
sp|P25344|STE50_YEAST/152-250	STE50_ORTHOLOG/L.klu/151-229	0.154
sp|P40086|COX15_YEAST/1-45	sp|P40416|ATM1_YEAST/1-84	0.111
sp|P40086|COX15_YEAST/1-45	sp|P43555|EMP47_YEAST/1-37	0.013

Table S7. Performance of different tools on synthetic IDRs derived from Cox15 N-terminus(41)

WT IDR:	Cox15		
Replaced IDR:	N1	P1	P2
Experiment (ground-truth)	✗	✓	✓
SHARK-dive	✓	✓	✓
DEDAL	✓	✓	✓
BLAST (BLOSUM62)	✗	✗	✗
HMMER	✓	✓	✗

Note: E-value threshold of <0.05 used for BLAST and HMMER, 0.5 for SHARK-dive, 0 for DEDAL homology classifier (42). Blue cells indicate concordant homology predictions to experimental results (dark blue).

Table S8. Reference proteomes obtained from UniProt 2022-05 Release

Unique proteome ID	Organism (common name)	Taxonomy ID	No. IDRs
UP000005640	Homo Sapiens (Human)	9606	69383
UP000000589	Mus musculus (Mouse)	10090	67570
UP000000625	Escherichia coli	83333	5207
UP000002311	Saccharomyces cerevisiae (Baker's yeast)	559292	14985
UP000006548	Arabidopsis thaliana (Mouse-ear cress)	3702	60185
UP000000437	Danio rerio (Zebrafish)	7955	89418
UP000000803	Drosophila melanogaster (Fruit fly)	7227	41643

Table S9. Version information of all tools and programs

Tool	Minimum Version	Other versions used
Python	3.6.5	3.7.4, 3.7.7
BLAST (makeblastdb, BLASTp)	2.13.0	
HMMER (pHMMER, HMMscan)	3.1b2	
MUSCLE	3.8.31	
MAFFT	7.453	
Catboost	1.0.0	
Numpy	1.15.0	1.19.5, 1.21.6
Biopython	1.71	1.75, 1.78, 1.80
Pandas	1.0.5	1.1.2, 1.1.3, 1.1.5, 1.3.5
Sci-kit Learn	0.19.1	0.23.1, 0.24.2, 1.0
Scipy	1.5.4	1.6.2, 1.7.1
Matplotlib	3.1.1	3.3.4
Pickle	4.0	
Alfpy	1.0.6	
R	3.6.3	
DECIPHER	2.14.0	
Tensorflow	2.12.0-dev20221213	
DEDAL	TF2.0 Saved Model (v3)	
CD-HIT	4.6	4.8.1

Supplementary Datasets

Dataset S1. Alignable-disorder dataset (Data_S1_alignable_disorder_dataset.zip).

Dataset S2. Unalignable-ortholog dataset (Data_S2_unalignable_ortholog_dataset.zip).

Dataset S3. Homology predictions of FUS PLD (aa 1-211) to IDRs in 7 model organisms (Data_S3_FUS-PLD_homology_pred.csv)

Dataset S4. Hyperparameter tuning results (see Data_S4_hyperparameters.csv)

Dataset S5. Alignment of Ded1p orthologs (Data_S5_DED1_ortholog_alignment.aln).

Dataset S6. Homology predictions of FUS RBD (aa 212-536) to IDRs in 7 model organisms (Data_S6_FUS-RBD_homology_pred.csv).

Supplementary References

1. UniProt Consortium, UniProt: the Universal Protein Knowledgebase in 2023. *Nucleic Acids Res.* **51**, D523–D531 (2023).

2. J. Mistry, *et al.*, Pfam: The protein families database in 2021. *Nucleic Acids Res.* **49**, D412–D419 (2021).

3. B. Mészáros, G. Erdös, Z. Dosztányi, IUPred2A: Context-dependent prediction of protein disorder as a function of redox state and protein binding. *Nucleic Acids Res.* **46**, W329–W337 (2018).

4. Z. Dosztányi, Prediction of protein disorder based on IUPred. *Protein Sci.* **27**, 331–340 (2018).

5. R. Grantham, Amino acid difference formula to help explain protein evolution. *Science* **185**, 862–864 (1974).

6. Z. Dosztányi, V. Csizmók, P. Tompa, I. Simon, The pairwise energy content estimated from amino acid composition discriminates between folded and intrinsically unstructured proteins. *J. Mol. Biol.* **347**, 827–839 (2005).

7. F. L, N. B, Z. Z, W. S, L. W, CD-HIT: accelerated for clustering the next-generation sequencing data. *Bioinformatics* **28**, 3150–3152 (2012).

8. DisProt in 2022: improved quality and accessibility of protein intrinsic disorder annotation. *Nucleic Acids Res.* **50**, D480–D487 (2022).

9. A. M. Altenhoff, *et al.*, OMA orthology in 2021: website overhaul, conserved isoforms, ancestral gene order and more. *Nucleic Acids Res.* **49**, D373–D379 (2021).

10. G. Hu, *et al.*, flDPnn: Accurate intrinsic disorder prediction with putative propensities of disorder functions. *Nat. Commun.* **12**, 4438 (2021).

11. K. Katoh, K. Misawa, K.-I. Kuma, T. Miyata, MAFFT: a novel method for rapid multiple sequence alignment based on fast Fourier transform. *Nucleic Acids Res.* **30**, 3059–3066 (2002).

12. A. Larsson, AliView: a fast and lightweight alignment viewer and editor for large datasets. *Bioinformatics* **30**, 3276–3278 (2014).

13. A. M. Waterhouse, J. B. Procter, D. M. A. Martin, M. Clamp, G. J. Barton, Jalview Version 2--a multiple sequence alignment editor and analysis workbench. *Bioinformatics* **25**, 1189–1191 (2009).

14. C. D. Livingstone, G. J. Barton, Protein sequence alignments: a strategy for the hierarchical analysis of residue conservation. *Comput. Appl. Biosci.* **9**, 745–756 (1993).

15. J. C. Lee, N. A. Rashid, Adapting normalized google similarity in protein sequence comparison. *Proceedings - International Symposium on Information Technology 2008, ITSim* **1**, 6–10 (2008).

16. B. B. Luczak, B. T. James, H. Z. Girgis, A survey and evaluations of histogram-based statistics in alignment-free sequence comparison. *Brief. Bioinform.* **20**, 1222–1237 (2019).

17. A. Zielezinski, *et al.*, Benchmarking of alignment-free sequence comparison methods. *Genome Biol.* **20**, 1–18 (2019).

18. F. Keul, M. Hess, M. Goesele, K. Hamacher, PFASUM: a substitution matrix from Pfam structural alignments. *BMC Bioinformatics* **18**, 293 (2017).

19. L. Prokhorenkova, G. Gusev, A. Vorobev, A. V. Dorogush, A. Gulin, CatBoost: unbiased boosting with categorical features. *arXiv [cs.LG]* (2017).

20. R. F. Doolittle, Similar amino acid sequences: chance or common ancestry? *Science* **214**, 149–159 (1981).

21. G. P. S. Raghava, G. J. Barton, Quantification of the variation in percentage identity for protein sequence alignments. *BMC Bioinformatics* **7**, 415 (2006).

22. J. M. Cherry, *et al.*, Saccharomyces Genome Database: the genomics resource of budding yeast. *Nucleic Acids Res.* **40**, D700-5 (2012).

23. T. Zarin, *et al.*, Proteome-wide signatures of function in highly diverged intrinsically disordered regions. *Elife* **8** (2019).

24. T. Zarin, C. N. Tsai, A. N. N. Ba, A. M. Moses, Selection maintains signaling function of a highly diverged intrinsically disordered region. *Proc. Natl. Acad. Sci. U. S. A.* **114**, E1450–E1459 (2017).

25. The Statistics of Sequence Similarity Scores. Available at: https://www.ncbi.nlm.nih.gov/BLAST/tutorial/Altschul-1.html [Accessed 10 May 2023].

26. R. C. Edgar, MUSCLE: multiple sequence alignment with high accuracy and high throughput. *Nucleic Acids Res.* **32**, 1792–1797 (2004).

27. V. Muñoz, L. Serrano, Elucidating the folding problem of helical peptides using empirical parameters. *Nat. Struct. Biol.* **1**, 399–409 (1994).

28. C. Iserman, *et al.*, Condensation of Ded1p Promotes a Translational Switch from Housekeeping to Stress Protein Production. *Cell* **181**, 818-831.e19 (2020).

29. M. Lindström, B. Liu, Yeast as a Model to Unravel Mechanisms Behind FUS Toxicity in Amyotrophic Lateral Sclerosis. *Front. Mol. Neurosci.* **11**, 218 (2018).

30. S. Ju, *et al.*, A yeast model of FUS/TLS-dependent cytotoxicity. *PLoS Biol.* **9**, e1001052 (2011).

31. J. Fassler, P. Cooper, *BLAST Glossary* (National Center for Biotechnology Information (US), 2011).

32. *Appendices* (National Center for Biotechnology Information (US), 2021).

33. S. Henikoff, J. G. Henikoff, Amino acid substitution matrices from protein blocks. *Proc. Natl. Acad. Sci. U. S. A.* **89**, 10915–10919 (1992).

34. M. O. Dayhoff, M. O. Dayhoff, R. M. Schwartz, Chapter 22: A model of evolutionary change in proteins. *IN ATLAS OF PROTEIN SEQUENCE AND STRUCTURE* (1978).

35. E. S. Wright, DECIPHER: harnessing local sequence context to improve protein multiple sequence alignment. *BMC Bioinformatics* **16**, 322 (2015).

36. E. Wright, Using DECIPHER v2.0 to analyze big biological sequence data in R. *R J.* **8**, 352 (2016).

37. R. Trivedi, H. A. Nagarajaram, Amino acid substitution scoring matrices specific to intrinsically disordered regions in proteins. *Sci. Rep.* **9**, 1–12 (2019).

38. S. F. Altschul, W. Gish, W. Miller, E. W. Myers, D. J. Lipman, Altschul et al.. 1990. Basic Local Alignment Search Tool.pdf. *Journal of Molecular Biology* [Preprint] (1990). Available at: http://dx.doi.org/10.1016/S0022-2836(05)80360-2.

39. S. R. Eddy, "HMMER User's Guide Biological sequence analysis using profile hidden Markov models" (2019).

40. R. D. Finn, J. Clements, S. R. Eddy, HMMER web server: Interactive sequence similarity searching. *Nucleic Acids Res.* **39**, 29–37 (2011).

41. B. Strome, K. Elemam, I. Pritisanac, J. D. Forman-Kay, A. M. Moses, Computational design of intrinsically disordered protein regions by matching bulk molecular properties. *bioRxiv* 2023.04.28.538739 (2023).

42. F. Llinares-López, Q. Berthet, M. Blondel, O. Teboul, J.-P. Vert, Deep embedding and alignment of protein sequences. *Nat. Methods* **20**, 104–111 (2023).
